# Risk factors for cervical cancer in women in China: A meta-model

**DOI:** 10.1177/1745506520940875

**Published:** 2020-08-12

**Authors:** Samuel Aballéa, Ekkehard Beck, Xiao Cheng, Nadia Demarteau, Xiao Li, Fangfang Ma, Mohamed Neine, Fang-Hui Zhao

**Affiliations:** 1 Creativ-Ceutical, Paris, France; 2 Public Health Department-Research Unit EA3279, Aix-Marseille University, Marseille, France; 3 Creativ-Ceutical, London, UK; 4 GSK, Wavre, Belgium; 5 Creativ-Ceutical Asia Limited, Hong Kong SAR, China; 6 Creativ-Ceutical, Beijing, China; 7 National Cancer Center/Cancer Hospital, Chinese Academy of Medical Sciences (CAMS) and Peking Union Medical College (PUMC), Beijing, China

**Keywords:** cancer risk, cervical cancer, China, meta-analysis, meta-model

## Abstract

**Objectives:**

Cervical cancer is a leading cause of cancer-related mortality in women in China. This analysis is a quantitative evidence synthesis pooling information about each cervical cancer risk factor.

**Methods:**

A meta-model was developed to estimate the risk of cervical cancer for a woman aged 18–85 years in Mainland China based on her risk profile at the time of assessment. The meta-model was built using findings of a systematic literature review that identified 21 case–control studies reporting data on 105 groups of cervical cancer risk factors in Chinese women. Extracted risk factors were ranked, and 17 were selected by Chinese clinical experts for inclusion in the meta-model. Risk equations were developed for each selected study. Predicted risks for each study were dependent on the risk profile under consideration and study-specific risks were pooled to an overall risk estimate using a random-effects meta-analysis. Sensitivity analysis was conducted using 100 artificial patient profiles (in the absence of patient data).

**Results:**

Predicted risks for the 100 profiles suggested that the model had good face validity and could differentiate between high and non-high cervical cancer risk profiles.

**Conclusion:**

This innovative meta-model approach assesses cervical cancer risk in Chinese women from a holistic perspective and could be adapted for other diseases and settings.

## Introduction

Cervical cancer (CC) is a major cause of cancer mortality among women in China, with an estimated 100,700 new cases (estimated incidence of 10.1–15.3 per 100,000 women) and 26,400 (estimated mortality rate of 2.59–2.76) deaths recorded in cancer registries in 2012.^1–4^ There may be considerable heterogeneity of CC incidence within China, with higher disease burden in some rural areas.^4,5^ Age-standardized incidence and mortality rates for CC in China both increased over the period from 1989 to 2008.^6^ Several risk factors have been associated with the acquisition of the human papillomavirus (HPV) and, thus, risk of CC, such as age and the number of sexual partners.^7,8^ However, there is no comprehensive evaluation of potential risk factors specific to women in China.^9^ A systematic review conducted to address this gap in the literature identified numerous risk factors statistically associated with CC in China. The main risk factors were socio-demographic (age and education level), life-style behavior (dietary consumption, smoking status, and personal hygiene), sexual behavior (number of partners (for the woman and her partner), number of marriages, age at sexual debut, and age at first marriage), gestational factors (age at first pregnancy, total number of pregnancies, and contraceptive method), and screening and disease history (cervical screening, gynecological diseases, family disease history, and other diseases).^10^ Persistent infection with HPV is a necessary cause of CC, detected in more than 99% of cases.^8^

Risk factors for disease are commonly evaluated using case–control studies, in which the frequency of suspected risk factors or other attributes is compared between people with the disease of interest (cases) and people without that disease (controls), providing odds ratios (OR) for the various factors. By aggregating results from multiple studies together, the risk of bias can be minimized and precision can be increased. The most common current approach to aggregating results uses meta-analysis methods.^11^ However, current meta-analysis techniques can only combine data from studies using similar variables and measures.^11^ They cannot be used when studies use different statistical models, different subsets of potential explanatory variables, or different transformations on included variables.^11^ Conventional meta-analysis can combine multiple measurements of the same thing (e.g. pooling ORs for the same risk factor), but cannot synthesize evidence related to multiple risk factors simultaneously.

The heterogeneity between studies in the systematic review on risk factors associated with CC in China meant that the risk factor data identified in the systematic review (i.e. OR) could not be combined, for example, because different data were reported (such as coefficient of risk equation vs OR) or different statistical models were used.^10^ Thus, the objective of this analysis was to explore a novel method of considering and combining information on multiple risk factors from several studies to predict the risk of CC for any woman’s profile and assess the overall contribution of each risk factor to the overall risk of CC. The technique chosen was to develop a meta-model, which combines several regression equations into a unified model to present the relationship between the overall estimated effect and the various risk factors in order to estimate the probability that an adult (aged 18–85 years) woman in Mainland China is at risk of CC based on her characteristics at the time of assessment.

## Methods

### Study overview

The analysis had three main steps. First, relevant data and risk factors were extracted from the case–control studies identified by a systematic literature review.^10^ Second, the risk factor information extracted from the case–control studies was reviewed by a panel of Chinese experts, who were asked to select the most important or most plausible risk factors for CC, based on their insights. Third, the final meta-model was developed based on the risk factors selected by the experts.

### Literature search and data extraction

The case–control studies identified in a previous systematic literature review^10^ were evaluated, and publications that did not come from Chinese core journals were removed. Core journals were defined as journals listed in the indexes of the Peking University Chinese Core Journal List (PKU; http://lib.nefu.edu.cn/attachment/20160428161107508.pdf), the China Science Citation Database (CSCD; http://sciencechina.cn/cscd_source.jsp), and the Chinese Social Sciences Citation Index (CSSCI; http://cssrac.nju.edu.cn/a/xwdt/zxdt/20170116/2805.html). An updated literature search was conducted on 6 July 2016 in five databases: Medline (Ovid MEDLINE In-Process & Other Non-Indexed Citations, Ovid MEDLINE Daily, Ovid MEDLINE, and Ovid OLDMEDLINE 1946 to Present) (English); EMBASE (via Ovid EMBASE) (English); CNKI (Chinese); Wanfang (Chinese); and CQVIP (Chinese), with a time frame of March 2014 to July 2016. Details of the search strategy are presented in Supplemental Appendix 1. Supplemental Appendix Figure 1 summarizes the systematic review and search update.

Studies were selected according to the inclusion and exclusion criteria summarized in Supplemental Appendix 2. Abstracts were screened by two independent reviewers, and any disagreements were resolved by consensus among the two reviewers and a third reviewer. During the full paper screening, the two independent reviewers also excluded publications which did not report any of the risk factors chosen by the experts at the expert meeting (see below).

Based on the results of the first round of sensitivity analysis of the meta-model, it was found that studies where either only one risk factor was reported or only one risk factor could be extracted significantly biased the model predictions. To reduce the risk of such bias, risk factor estimates from a specific study were included in the model only if risk factor estimates could be extracted for at least two risk factors. Risk factor estimates were, therefore, considered for inclusion in the final model if: the OR (OR_*j,k*_), relative risk (RR) (RR_*j,k*_) or regression coefficient $\left(\sigma_{j,k}\right)$ for risk factor *j* in study *k* was reported; the standard error (SE), variance, confidence interval (CI), or *p*-value of the OR or RR or any other data was reported from which the SE around the log-OR of the risk factor $\sigma_{j,k}$ could be derived; and at least two risk factors were reported in the study for which all risk factor estimates fulfilled the above two criteria. If both the univariate and multivariate OR, RR, or regression coefficient for a specific risk factor was reported, only the multivariate OR, RR, or regression coefficient was included in the model. Due to the limited availability of data, if no multivariate OR, RR, or regression coefficient was reported, the univariate estimate was included in the model. A risk factor was defined as the variable used in the regression analysis (e.g. “age at sexual debut”), and risk factor levels were defined as the categories used for that variable (“age <17 years,” “age 17–19 years,” etc.). Prevalence estimates (estimates of the prevalence of the risk factor in the control group) were also extracted from each study. Data extraction was performed by one reviewer, followed by quality control by a second reviewer. Any disagreements were resolved by consensus. The extracted data were further reviewed by the project manager when the extracted data were integrated into the statistical model.

### Analysis of extracted data

The original systematic review grouped risk factors into 6 main categories and 44 sub-categories.^10^ For the present analysis, risk factors were further stratified into more detailed categories to allow meaningful comparison of risks and prevalence. This resulted in 105 different groups of risk factors, which we refer to in the remainder of the article as 105 “risk factors” for simplicity (Supplemental Appendix Figure 2). For each of these risk factors, the reporting frequency, the pooled OR, and the pooled prevalence were calculated, to inform the choice of risk factors to keep in the model. The risk factors from the original systematic review were then reviewed at a meeting of experts as described in section “Expert meeting,” and the experts selected the most important risk factors to be included in the final meta-model, as summarized in Supplemental Appendix Figure 2.

#### Imputation of missing data

##### Imputation of OR and corresponding SE

To obtain the pooled OR for various risk factors, missing data for the corresponding risk factor–level estimates had to be imputed. Because all underlying studies were case–control studies, a value reported as an RR was considered to be incorrectly designated and instead assumed to be the OR. This assumption was made because it is not feasible to estimate RR directly from a case–control study, and for rare outcomes such as CC, the RR and OR are similar. If only the regression coefficient $\sigma_{j,k}$ was reported, we derived the OR_*j,k*_ using the unbiased mean estimate^12^

$${\mathrm{OR}}_{j,k}=exp\left(\mu_{j,k}+\frac{\sigma_{j,k}^{2}}{2}\right)$$

If the SE of the log-OR of a risk factor estimate, that is,$\sigma_{j,k}$, was not reported, we imputed this value based on the available data and assuming the log-OR to be normally distributed. If the 95% CI of the OR or RR was available, we computed $\sigma_{j,k}^{2}$ assuming the lower and upper bounds of the 95% CI given to be

$$95\%\;\;\mathrm{CI}:\left[\begin{array}{l} \exp\left(\mu_{j,k}+\frac{\sigma_{j,k}^{2}}{2}-1.96\;\sigma_{j,k}\right);\\ \exp\left(\mu_{j,k}+\frac{\sigma_{j,k}^{2}}{2}+1.96\;\sigma_{j,k}\right) \end{array}\right]$$

If the *p*-value was the only available information about the variability of the OR, we applied a procedure proposed by Altman and Bland^13^ to derive $R_{0k}$ from the *p*-value as follows

$$z_{j,k}=-0.862+\sqrt{0.743-2.404\;\log\left(p{\mathrm{-value}}_{j,k}\right)}$$

$$\sigma_{j,k}=\left|\frac{\mu_{j,k}}{z}\right|$$

If the OR, the χ²-statistic, and the total number of cases and controls were reported but no other information about the variability of the OR was provided, we calculated the entries of the contingency table numerically by solving a system of four equations. The contingency table entries were as follows: *a* = cases exposed, *b* = controls exposed, *c* = cases not exposed, and *d* = controls not exposed. Solving the following system of four equations with the provided input on the left side of each equation, we determined each entry of the contingency table

Totalcontrols=b+d

Totalcases=a+c

$$\mathrm{RR}=\frac{a/\left(a+b\right)}{c/\left(c+d\right)}$$

$$\chi^{2}\mathrm{-statistic}=\frac{{\left(ad-bc\right)}^{2}\left(a+b+c+d\right)}{\left(a+b\right)\left(c+d\right)\left(b+d\right)\left(a+c\right)}$$

Based on the solutions to this equation system, we then calculated the variance of the log-OR ($N_{k}$) assuming the log-OR to be normally distributed. Using the solutions for *a, b, c*, and *d*, we then computed the variance of the log-OR as follows

$$\sigma_{j,k}^{2}=\frac{1}{a}+\frac{1}{b}+\frac{1}{c}+\frac{1}{d}$$

##### Imputation of prevalence

If the prevalence of a specific risk factor level was not reported in the publication, it was imputed by the average prevalence of the corresponding risk factor levels reported in the other case–control studies. If this procedure was not applicable, a targeted literature search was performed to obtain a population estimate based on Chinese data sources.

#### Pooled OR and prevalence

To provide a meaningful ranking among the 105 risk factors based on the magnitude of the effect, ORs corresponding to the non-reference risk factor levels within each risk factor were pooled using a weighted average approach where the weight was the inverse of the variance corresponding to the OR. To allow a meaningful comparison between risk factors, the inverse of the OR was considered if the originally reported OR was <1. Similarly, pooled prevalence estimates of the non-reference risk factor levels within each risk factor were derived by calculating a weighted average prevalence.

### Expert meeting

An expert meeting was held in Beijing on 22 April 2016 to select the most important risk factors to be included in the final meta-model based on the extracted and analyzed risk factors. Six clinicians and one statistical expert from hospitals in Beijing participated in the meeting. The process of the meeting is summarized in Supplemental Appendix 3. Experts were also presented with and asked to discuss the methodological concept of the meta-model.

After discussing the input data, including the ranking of risk factors, and the statistical model with the experts, an update of the previous literature search was conducted to seek estimates for missing risk factors, as described above. A separate search was conducted for population-level estimates for the prevalence of risk factor levels since the experts considered population-based prevalence estimates to be more plausible than prevalence estimates extracted from the case–control studies.

As detailed above, the development of the meta-model required estimates of the prevalence of different risk factor levels, as well as ORs or RRs associated with those risk factor levels. The prevalence search used a system of tiers to differentiate and prioritize the data sources. Tier 1 data were from public health databases providing Chinese population-based estimates. Tier 2 data were from large cohort studies and public surveys. Tier 3 data were from grey literature. Tier 4 data were the control group prevalences in the original case–control studies from which the risk factor estimates had been derived. Finally, Tier 5 data were from non-Chinese data sources, searched using the same approach as the first three tiers; data were prioritized based on geographical and cultural proximity to China. If no result was found after searching all five tiers, the risk factor was excluded from the model. For more details, see Supplemental Appendix 4.

### Development of meta-model

**Figure 1.**
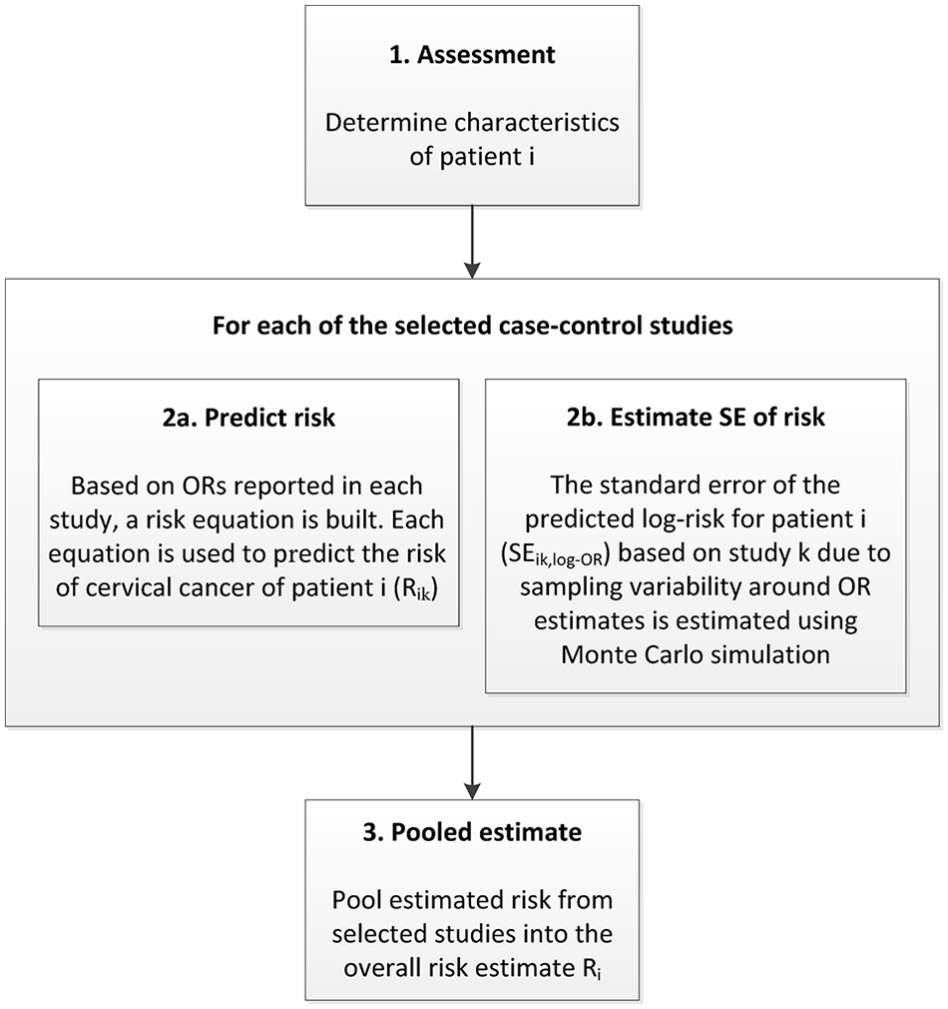
Overview of the structure of the meta-model. OR: odds ratio; SE: standard error.

Using the imputed values and the final list of risk factors selected by the experts, the meta-model was established to estimate the probability of a Chinese woman to have CC at the time of the assessment. Figure 1 provides an overview of the model structure.

First, the patient’s risk factor profile needs to be determined (step 1). Second (step 2a), the study-specific risk of CC is calculated based on the patient’s risk factor profile and a risk equation incorporating the OR and prevalence of each risk factor in each study. Then the SE of the log-risk is calculated using Monte Carlo simulation (step 2b). Finally, the overall risk of the patient for CC is calculated using a random-effects meta-analysis (step 3) (Figure 1).

#### Individual patient risk

For each study *k*, the risk of CC for the reference patient (i.e. in whom all risk factors were at reference levels), $R_{0}$ is estimated using the following equation

$$R_{0k}=\frac{R_{0}}{Exp\left(\sum_{1}^{N_{k}}\alpha_{j}\;\log\left({\mathrm{OR}}_{jk}\right)\right)}$$

where *j* is the risk factor; *k* is the study; $\alpha_{j}$ is the number of risk factors in the study *k*; ${\mathrm{OR}}_{jk}$ is the probability of having CC in Chinese women aged 18–85 years in 2016, which is 0.000094;^14^ $R_{0}$ is the value or prevalence of each level of risk factor *j* in the general population (Chinese women aged 18–85 years); and${\mathrm{OR}}_{jk}$ is the OR for each level of risk factor *j* in study *k*.

The overall population-level risk $R_{ik}$ is divided by the RR between the average Chinese woman and the reference patient in each study. This RR is calculated as the product of ORs geometrically weighted by the population prevalence of the risk factors.

This baseline risk is then weighted with the patient-specific risk profile *i* to calculate the patient- and study-specific risk of CC using the following equation

$$R_{\mathrm{ik}}=\left(\overset{N_{k}}{\underset{j}{\prod }}{\mathrm{OR}}_{jk}\right)*R_{0k}$$

where $\left(R_{i}\right)$ is the OR for the level of risk factor *j* reported by patient *i* in study *k*.

#### Monte Carlo simulation

To pool the patient- and study-specific risks $R_{ik}$ in order to obtain the overall risk for a patient to have CC $R_{ikn}$, each study-specific risk was weighted by the within- and between-study variance. Within-study variance (the variability around the risk $\sigma_{i,k}$) was obtained using the Monte Carlo simulation. At each replication, an OR estimate and a prevalence estimate for each risk factor were sampled, and the study-specific risk $\left(\log(R_{ik})\right)$ was calculated as described above. The number of replications was 10,000. When sampling the OR and prevalence estimates, we assumed the log-OR to be normally distributed and the prevalence to be beta-distributed. As the variability around prevalence estimates was usually unknown, a coefficient of variation of 0.1 was assumed for all prevalence estimates. This was deemed a conservative choice, as the true coefficients of variation were expected to be smaller than this. The standard error $\left(\log(R_{ikn})\right)$ of the log-study risk $\sigma_{i,k}$ was calculated as the sample standard deviation of the simulated log-study risks $R_{i}$. The resulting $\sigma_{i,k}^{2}$ was used in the random-effects meta-analysis to calculate the overall risk $\tau_{i}^{2}$.

#### Random-effects meta-analysis

A random-effects model was chosen because of the heterogeneity across studies observed in the systematic review.^10,12^ The random-effects model considered both the variance within studies ($R_{i}$) and the variance between studies ($R_{ik}$) to determine the overall risk $R_{i}$. The variance within studies relates to the log-study risk, and thus the random-effects model aggregated the transformed log-risks of each study to determine an overall log-risk. Following the aggregation of the log-transformed study-specific risks $R_{ik}$, a back transformation was applied to obtain the overall risk $R_{i}$. For more details, see Supplemental Appendix 5.

The meta-model was implemented in *Microsoft Excel* using both Excel spreadsheets and Visual Basic for Applications (VBA).

### Model validation and patient profiles

Verification of the meta-model was performed by double-checking implemented formulas and input data, and recalculation of estimates. Face validity (the ability of the model to make reasonable predictions given changes to the input data) was tested using artificially created patient profiles, in the absence of actual patient-level data. An initial test was conducted using eight hypothetical patient profiles, created to provide realistic representations of eight Chinese women and to reflect some known risk factors of CC, such as age at sexual debut, number of pregnancies and deliveries, menopause, cervical screening, and smoking. The eight patient profiles are presented in Table 1. Further assessment was performed by conducting a sensitivity analysis. A full validation of the model including full predictive validation was not performed due to lack of actual patient-level data.

**Table 1.** Eight hypothetical patient profiles with 17 risk factors for cervical cancer and predicted individual risk for each profile.

No.	Risk factors		Patient 1	Patient 2	Patient 3	Patient 4	Patient 5	Patient 6	Patient 7	Patient 8
1	Age at sexual debut		19	20	16	19	18	21	20	19
2	Number of sexual partners	Life-time number of sex-partners	2	2	1	3	1	1	3	1
		Number of non-marital partners	1	1	0	1	0	0	1	1
3	HPV infection		Yes	No	Yes	No	No	No	Yes	No
4	Cervical/gynecological screening history (no. of negative smears)		4	NA	NA	1	NA	2	NA	NA
5	Time between screenings (last negative smear)		2	NA	NA	NA	NA	1	NA	NA
6	Age at first pregnancy		21	21.5	NA	27	18	25	20	NA
7	Age at first delivery		22	22.5	NA	28	19	26	21	NA
8	Number of pregnancies		1	2	NA	1	3	1	2	NA
9	Number of deliveries		1	1	NA	1	2	1	2	NA
10	Contraception methods	IUD use	No	No	No	No	Yes	Yes	No	No
		Condom use	Yes	No	Yes	No	No	No	No	No
11	Years on contraception (IUD use)		NA	NA	NA	NA	15	9	NA	NA
12	Menopause		No	No	No	No	Yes	No	Yes	No
13	Poor sexual hygiene (washing or not before sex)		Yes	Yes	No	No	No	Yes	No	No
14	Smoking (Yes/No, cigarette index)^a^		50	250	No	No	No	50	No	No
15	Second-hand smoking (Yes/No)		Yes	No	Yes	No	Yes	Yes	Yes	No
16	Educational level (5 college or above, 1 below primary school)		1	5	3	4	1	4	3	1
17	Occupations (Intellectual job—Yes/No)		No	No	Yes	No	No	Yes	No	Yes
Individual predicted risk of cervical cancer										
Individual predicted risk *R*_*i*			0.078%	0.016%	0.049%	0.011%	0.020%	0.005%	0.115%	0.009%
95% confidence interval			(0.027–0.219)	(0.009–0.031)	(0.025–0.098)	(0.007–0.015)	(0.013–0.031)	(0.004–0.008)	(0.035–0.378)	(0.007–0.012)
Population-level incidence *R*_0			0.009% (ratio = 8.28)	0.009% (ratio = 1.72)	0.009% (ratio = 5.23)	0.009% (ratio = 1.12)	0.009% (ratio = 2.09)	0.009% (ratio = 0.57)	0.009% (ratio = 12.27)	0.009% (ratio = 1.00)

HPV: human papillomavirus; NA: not applicable; IUD: intra-uterine device.

a Cigarette index (the number of cigarettes per day multiplied by the number of years of smoking).

### Sensitivity analysis

Sensitivity analysis was conducted by varying input parameters and assessing the change in the predicted risk. We measured accuracy, defined as the (absolute) deviation of the predicted risk in a scenario from the predicted risk in the base-case, and precision, defined as the deviation of the relative width of the 95% CI corresponding to the predicted risk from the relative width of the 95% CI in the base-case.

The impact of risk factors and studies on the overall risk was assessed by removing one risk factor or one study at a time and re-running the model, and then comparing the results with the base-case results to assess the impact of a single risk factor or single study on accuracy and precision. The impact of changing the coefficient of variation from the base-case value of 0.1 or the number of Monte Carlo simulations from 10,000 was assessed by changing the values and comparing the results with the base-case.

A rigorous analysis of the meta-model requires a sufficiently large set of patient profiles that are representative of the target population. In the absence of real patient-level data, we generated 100 patient profiles with an algorithm taking into account high-level dependencies between risk factors (e.g. the dependencies between the risk factors “age at sexual debut” and “age at first delivery” ensured that a created profile would have a first delivery at least 9 months after sexual debut). The algorithm further sampled the risk factor estimates for each patient profile based on the population prevalence of each risk factor to ensure a representative sample.

## Results

### Initial literature review and expert meeting

**Figure 2.**
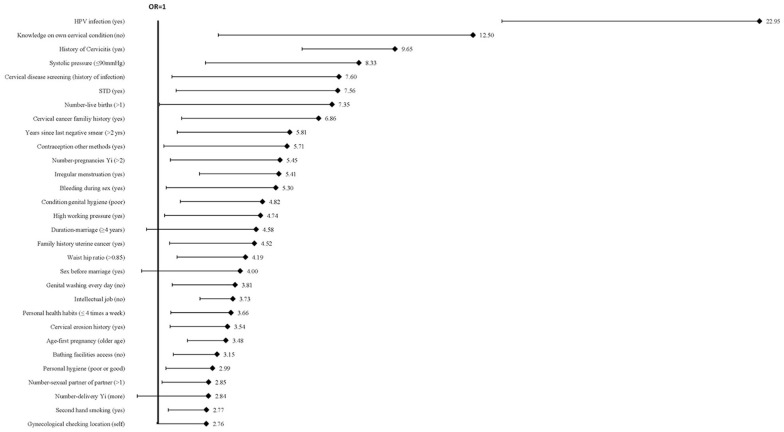
Top 30 risk factors among all risk factors (before the selection of risk factors at the expert meeting) ranked by odds ratio (OR). The error bar indicates the corresponding lower bound of the confidence interval (CI). The vertical line indicates OR =1. HPV: human papillomavirus; STD: sexually transmitted disease.

The initial literature review provided information on 507 risk factors estimates. Some of these were similar risk factors which were described using different terms across studies. Therefore, they were grouped into 105 risk factor categories, referred to in this article as 105 risk factors. Figure 2 shows the top 30 risk factors ranked by decreasing OR, with the corresponding value of the lower bound of the CI indicated by the error bars. The five most frequently reported risk factors were smoking (nine studies), age at sexual debut (nine studies), number of deliveries (nine studies), HPV infection (eight studies) and number of pregnancies (seven studies) (Supplemental Appendix 6 and Supplemental Appendix Figure 3).

Figures 4 and 5 in Supplemental Appendix 6 show the 30 risk factors with the highest and lowest prevalence, respectively. Risk factors with very high or very low prevalence are less likely to discriminate well between women.

The experts recommended including risk factor estimates from studies published in Chinese core journals or international journals, and obtaining population-level estimates for the prevalence of risk factors. The experts recommended the inclusion of 18 risk factors.

### Updated literature search and meta-model

The final meta-model consisted of 140 risk factor levels reported in 11 studies (Supplemental Appendix Figures 1 and 2) corresponding to 17 out of 18 risk factors recommended for inclusion based on discussion of the literature review results with clinical experts. For the recommended risk factor “history of cervical treatment,” no study reporting this risk factor could be identified in the updated search, and thus this risk factor was not included in the final meta-model. Prevalence estimates were obtained for all 140 risk factor levels. Table 2 lists the 17 risk factors included in the final meta-model and presents information on the prevalence data available for each. Supplemental Appendix 7 presents the results data for each of the risk factors in the final meta-model. Supplemental Appendix 8 summarizes the characteristics of the studies included.

**Table 2.** Prevalence data for the risk factors included in the final meta-model.

Risk factor	Prevalence estimates obtained (yes or no)	Tier	Direct estimate or calculation	Comments
Age at sexual debut	Yes	Tier 1/2	Calculation	The average age at sexual debut calculated based on Kaplan–Meier curves of sexual debut among rural and urban women in Mainland China. The weighted average Kaplan–Meier curve was calculated using population estimates of rural and urban women
Number of sexual partners	Yes	Tier 2	Direct estimate	
Cervical/gynecological screening history	Yes	Tier 4	Direct estimate	In the absence of values, the original prevalence estimates of the underlying case–control study were used
Time between screenings (especially the time since the last cervical screening)	Yes	Tier 2	Calculation	Uniform distribution within broad screening intervals was assumed to derive the prevalence estimates for different durations between screenings
HPV infection	Yes	Tier 2	Direct estimate	
Number of pregnancies	Yes	Tier 1/2	Calculation	Calculation based on natural infertility rate of 17.14% and distribution of the number of pregnancies
Number of deliveries	Yes	Tier 1	Calculation	The number of women with live births as reported by the National Bureau of Statistics of China divided by total women ever with live births
Age at first pregnancy	Yes	Tier 1/2	Direct estimate	
Age at first delivery	Yes	Tier 1	Calculation	The number of women with first live birth in different ages reported by the National Bureau of Statistics of China divided by total women with first live birth
Contraception methods	Yes	Tier 1/2	Calculation	Average of two references
Years on contraception	Yes	Tier 2	Direct estimate	
Menopause (Yes/No)	Yes	Tier 1/2	Calculation	Probability/percentage of Chinese women aged 18–85 years who have already experienced menopause based on age distribution of menopause and age distribution in population
Smoking (Yes/No, years of smoking)	Yes	Tier 1	Direct estimate	
Second-hand smoking (Yes/No)	Yes	Tier 2	Calculation	An estimate of second-hand smoke among both sexes and the fractions of non-smoking females (97.6%) and males (47.1%) were used to derive the sex-specific distribution of non-smokers in women and men
Poor sexual hygiene (i.e. washing or not before sex)	Yes	Tier 2	Direct estimate	
Educational level	Yes	Tier 1	Calculation	The number of women with various educational level reported by the National Bureau of Statistics of China divided by the total number of Chinese women
Occupations	Yes	Tier 1	Calculation	The number of women with various occupations reported by the National Bureau of Statistics of China divided by the total number of Chinese women

HPV: human papillomavirus.

### Meta-model results, initial analysis

The model-predicted risk *R_i_* for the three HPV-positive patient profiles (profiles 1, 3, and 7) was significantly higher than the average predicted risk among all eight profiles, the overall population incidence, and the model-predicted risk of the HPV-negative patient profiles (Table 1).

### Sensitivity analysis

**Figure 3.**
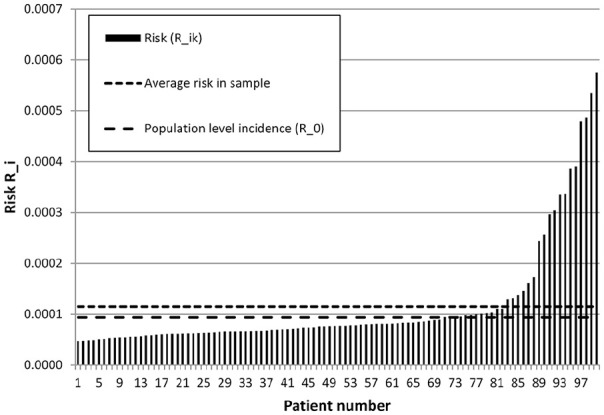
Predicted risk for 100 patient profiles in the base-case analysis with all risk factors and all studies included.

Figure 3 shows the model-predicted risk in the base-case for each of the 100 patient profiles generated by the algorithm. All 17 risk factors and all 11 studies were included in this analysis. The average predicted risk across the 100 patient profiles was slightly higher than the overall population incidence *R*_0_ (Figure 3). The predicted risk varied considerably between the 100 patient profiles, ranging from 0.5 times *R*_0_ to 6.12 times *R*_0_ (Figure 3). Eighteen of the 100 profiles had a predicted risk higher than the average of the sample. Of these 18 profiles, 12 were HPV-positive.

**Figure 4.**
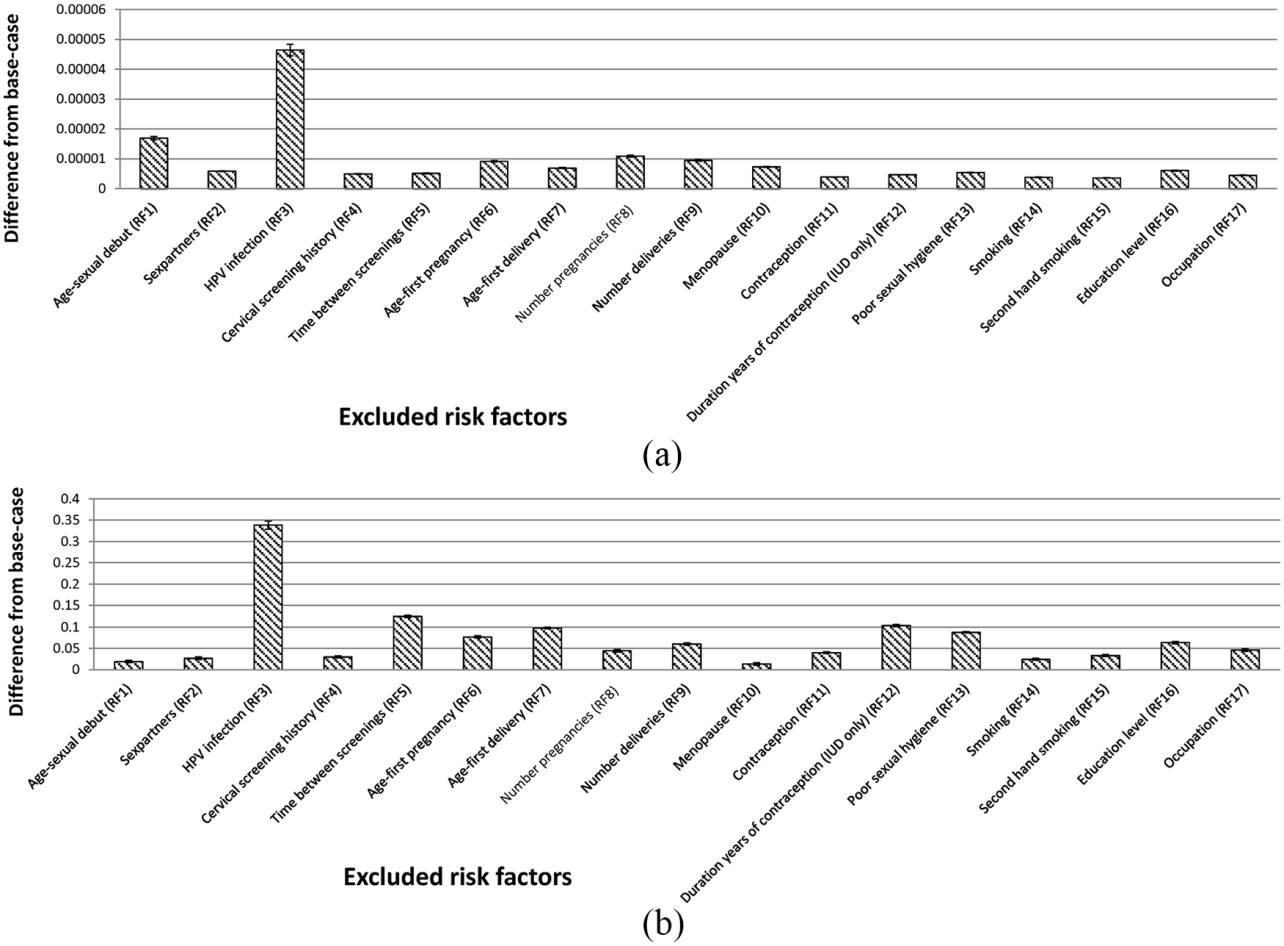
Impact of risk factors: (a) absolute difference in predicted risk compared with base-case (model accuracy); (b) difference in relative width of 95% confidence interval (CI) compared with base-case (model precision). IUD: intra-uterine device; HPV: human papillomavirus; RF: risk factor.

Figure 4(a) shows the impact on the model accuracy of removing risk factors from the analysis one at a time, presented as the average of the absolute difference between the predicted risk in the base-case and the predicted risk without the specified risk factor. Removing the risk factor “HPV infection” had the largest impact on the accuracy of the predicted risk. Other risk factors with a substantial impact included the sexual behavior risk factor “age at sexual debut” and gestational risk factors such as “age at first pregnancy/delivery,” “number of pregnancies/deliveries,” and “menopause.”

Figure 4(b) shows the impact on the model precision of removing risk factors from the analysis, presented as the difference in the relative width of the 95% CI compared with the base-case. A positive difference indicates that the relative width of the CI was smaller without the risk factor. The risk factor “HPV infection” had the largest impact on precision. Gestational risk factors such as “age at first pregnancy/delivery” and “number of deliveries” that had an impact on accuracy (Figure 4(a)) also had notable impacts on precision (Figure 4(b)).

Supplemental Appendix 9 shows the results of the sensitivity analysis in which studies were removed from the analysis one at a time. Removing a single study had a smaller impact on accuracy than removing a risk factor, with a deviation of no more than 20% of the predicted risk in the base-case (Supplemental Appendix Figure 6 and Supplemental Appendix 9). The impact of removing a single study increased with the number of risk factors in the study.

Removing a single study generally decreased model precision (Supplemental Appendix Figure 7 and Supplemental Appendix 9). This would be expected, as increasing the amount of data is generally assumed to improve the precision of a model. However, two studies increased model precision when removed (Supplemental Appendix Figure 7, Supplemental Appendix 9). They reported large numbers of risk factors and high ORs, which increased the uncertainty in predicted risk.

Altering the number of Monte Carlo simulations did not result in a significant change in model precision. When the coefficient of variation around prevalence estimates increased or decreased, the average width of the 95% CI around predicted risks also increased or decreased.

## Discussion

We developed a meta-model to predict the risk of CC for a woman in Mainland China aged 18–85 years at the time of assessment, based on individual patient characteristics. The meta-model was developed from data published in 11 case–control studies of risk factors associated with CC in Chinese women. The risk factor with the highest average OR for CC was HPV infection, and the risk factors reported in the highest number of studies were smoking, age at sexual debut, and number of deliveries (nine studies each). Second-hand smoking was reported in a smaller number of studies than smoking (two studies), although both had similar pooled OR when ranking the original 105 risk factors presented to the experts (section “Pooled OR and prevalence”) (2.77 for second-hand smoking vs 1.67 for smoking index, 2.06 for smoking and 3.18 for cigarette smoking, respectively). In the initial analysis, the model-predicted risk *R_i_* for the three HPV-positive patient profiles was significantly higher than the average predicted risk across all the profiles, the overall population incidence, and the model-predicted risk of the HPV-negative patient profiles, indicating that the model could distinguish between patient profiles with a high or non-high risk of CC. In a subsequent analysis using 100 artificial patient profiles, 12 of the 18 profiles with a predicted risk higher than the average for the sample were HPV-positive, again indicating that the model was able to distinguish between patient profiles with a high or non-high risk of CC. Sensitivity analysis found that the risk factor with the largest impact on predicted risk was HPV infection, followed by age at sexual debut and gestational risk factors. These results indicate that the model has acceptable face validity, as risk factors such as HPV infection, age at sexual debut, and gestational factors are included in other tools for assessing CC risk such as the Harvard Disease Risk Index.^15^

Our study has several strengths. We excluded studies not published in the Chinese Core Journal List (see section “Literature search and data extraction”) in the updated systematic literature review to maximize the quality of the data included. Furthermore, our meta-model was explicitly designed to aggregate risk factor estimates from studies with different ranges for categorical and binary outcomes of risk factor variables and with different combinations of risk factors. Combining data from such disparate sources would not have been possible when aggregating ORs in a conventional meta-analysis. The ability to combine data from diverse studies is a key benefit of the meta-model approach, making it possible to combine data even from the highly heterogeneous studies identified in the systematic review. The meta-model was able to handle different definitions of ranges of risk factors because it used study-specific baseline risk multiplied by the OR. In addition, our model included both expert knowledge and risk factors reported in the literature as input data and could potentially be applied to predict CC risk in all Chinese women. This is a potential advantage over other predictive models for CC, which typically rely on a specific set of patient data and risk factors.^16,17^ Finally, our model assesses CC risk based on a limited list of risk factors rather than the risk of cervical pre-cancer based on CC screening results, such as the analysis reported by Rothberg et al.^18^

The meta-model was also able to combine both adjusted and non-adjusted risk factor estimates. However, the use of both univariate and multivariate risk factor estimates could potentially bias predicted risks. Some of the 17 risk factors chosen by the experts and included in the final model are likely to depend on each other, such as the number of pregnancies and the number of births. Using only univariate (unadjusted) estimates for two such dependent risk factors could lead to an over-estimation of the risk estimated from that study. If sufficient data were available, it would be preferable to include only multivariate estimates or to include only independent risk factors from a study reporting univariate estimates for several risk factors. This could be a potential area for further research.

The current analysis also has limitations. First, we had to use artificially created patient profiles to validate the model, because actual patient-level data were not available. Further validation of the model using actual patient data would be valuable.^19,20^

Second, the underlying studies were conducted between the 1970s and the 2010s, and the model assumed that these estimates were equally applicable to the current population. However, economic and societal changes over the past four decades are likely to have changed actual health and healthcare seeking behavior over time, and any such changes are not addressed in the current analysis.

Third, the woman’s age was not considered as a single risk factor in the meta-model, even though the risk of CC is known to be strongly correlated with the age of the woman and is highest in Chinese women aged 45–49 years.^14^ This was because none of the included studies reported OR estimates for age, although some^21–23^ adjusted OR estimates for age. Some of the risk factors in the final meta-model, such as menopause, included age indirectly. Age of exposure, which can influence the risk of CC,^24^ was also indirectly considered in the meta-model through risk factors such as age at sexual debut and age at first pregnancy. It would be possible to include age as a single risk factor in the model by adjusting the population-level incidence *R*_0_ in the baseline risk using an age factor. However, the effect of age would be to some extent double-counted.

Fourth, data on OR estimates and prevalence that met the quality criteria of the updated literature search were limited. Some studies were excluded and risk factor data were not extracted from others due to the poor quality of data reporting. The potential loss of information could have biased the accuracy and precision of the model predictions.

Furthermore, detailed information on risk factors was not provided in each study considered in the meta-model. For HPV-type infection, Ye et al.^25^ did not report on the specific HPV type infection in comparison with the five other studies which reported OR estimates for specific types of cancerous HPV infections. This could have resulted in biased estimation of the predicted risk and of the impact of the risk factor HPV infection on accuracy and precision. However, this would most probably have had a limited impact, as only Ye et al.^25^ (out of six studies) did not report detailed OR estimates. The OR reported by Ye et al.^25^ was also comparable to other ORs reported in the remaining included studies.

Potential areas for future research include validating the model with actual patient data. This could be done using cross-sectional patient data from a cohort study or a randomized clinical trial, comparing the model-predicted risk for individual patients with the predicted risk of the study-specific risk equation derived from a multivariate regression analysis on the cross-sectional data set. If data on eventual outcomes were available (i.e. whether each patient later went on to develop CC), a comparison of this data set with the model-predicted risk would also permit assessment of the predictive accuracy of the model. The current sensitivity analysis and validation presented here show whether the risk moves in the expected direction when a risk factor is introduced into a woman’s profile. It can also assess whether risk estimates are more sensitive to factors known to have higher impact (e.g. HPV). However, it does not tell us whether the absolute risk estimates provide an accurate representation of the true risk.

This methodology could be applied to any disease for which risk factors have been investigated in multiple case–control studies. It could also be extended to incorporate prospective cohort studies.

The meta-model described here offers an innovative approach to predicting the risk of CC in Chinese women. It integrates risk factor data published in the literature with expert knowledge and could be applied to predict CC risk in all Chinese women rather than being restricted to a specific set of patients. The meta-model approach is better able to analyze and synthesize risk factors and corresponding ORs across studies than conventional meta-analysis with single ORs. As the methods developed in this analysis can pool predictive equations from different studies using different sets of definitions of risk factors, it could also be applied to other disease areas where risk factors have been investigated using case–control studies or prospective cohort studies.

## Supplemental Material

Aballéa_et_al_Supplementary_file_Clean – Supplemental material for Risk factors for cervical cancer in women in China: A meta-model Click here for additional data file. Supplemental material, Aballéa_et_al_Supplementary_file_Clean for Risk factors for cervical cancer in women in China: A meta-model by Samuel Aballéa, Ekkehard Beck, Xiao Cheng, Nadia Demarteau, Xiao Li, Fangfang Ma, Mohamed Neine and Fang-Hui Zhao in Women’s Health
