# Supplementary material for: Risk factors for cervical cancer in women in China: A meta-model
Source: Womens Health (Lond). 2020 Aug 12;16:1745506520940875. doi: 10.1177/1745506520940875 (PMC7469728; doi:10.1177/1745506520940875)
Supplement: Aballéa_et_al_Supplementary_file_Clean – Supplemental material for Risk factors for cervical cancer in women in China: A meta-model [file Aballea_et_al_Supplementary_file_Clean.pdf]

Risk factors for cervical cancer in women in China: a meta-model

Appendices

Appendix 1. Search strategy

<u>Medline</u> via Ovid (Epub Ahead of Print, In-Process & Other Non-Indexed Citations, Ovid MEDLINE Daily and Ovid MEDLINE 1946 to Present); year 2014 to 6 July 2016. Search run on 6 July 2016.

#	Search terms	Hits
1	EXP Uterine Cervical Neoplasms/	65,808
2	((cervical OR cervix) ADJ3 (neoplasm$1 OR cancer$1)).TI,AB.	42,558
3	OR/1-2	78,778
4	EXP Risk Factors/	650,712
5	EXP Algorithms/	245,343
6	((risk ADJ1 factor$1) OR predictive OR predictivit$3 OR prediction$1 OR algorithm$1 OR epidemiol* OR determinant$1).TI,AB.	1,417,858
7	OR/4-6	1,938,736
8	EXP China/	123,459
9	(China OR Chinese).TI,AB.	226,080
10	OR/8-9	264,590
11	3 AND 7 AND 10	389
		102

<u>Embase</u> via Ovid; year 2014 to 6 July 2016. Search run on 6 July 2016.

#	Search terms	Hits
1	EXP Uterine Cervix Cancer/	73,657
2	((cervical OR cervix) ADJ3 (neoplasm OR neoplasms OR cancer OR cancers)).TI,AB.	53,461
3	OR/1-2	90,161
4	EXP Risk Factor/	756,078
5	EXP Algorithm/	223,043
6	((risk ADJ1 (factor OR factors)) OR predictive OR predictivity OR predictivities OR prediction OR predictions OR algorithm OR algorithms OR epidemiology OR epidemiologies OR epidemiologic OR epidemiological OR determinant OR determinants).TI,AB	1,782,203

#	Search terms	Hits
7	OR/4-6	2,188,894
8	EXP China/	131,615
9	EXP Chinese/	39,806
10	(China OR Chinese).TI,AB	282,206
11	OR/8-10	323,226
12	3 AND 7 AND 11	382
13	Limit 12 to YR="2014 - 2016"	117

CNKI; year 2014 to 6 July 2016. Search run on 6 July 2016.

#	Search terms	Hits
1	(SU='宫颈癌' OR SU='子宫颈癌') AND (KY='危险度' OR KY='危险因素' OR KY='危险性因素' OR KY='风险因素' OR KY='相关因素' OR KY='保护因素' OR KY='影响因素'OR KY='高危因素' OR KY='协同因素' OR KY='预期' OR KY='预测' OR TI='危险度' OR TI='危险因素' OR TI='危险性因素' OR TI='风险因素' OR TI='相关因素' OR TI='保护因素' OR TI='影响因素' OR TI='高危因素' OR TI='协同因素' OR TI='预期' OR TI='预测') NOT (TI='预后' OR TI='复发' OR TI='转移')	261

Wanfang Data; year 2014 to 6 July 2016. Search run on 6 July 2016.

#	Search terms	Hits
1	((T=(宫颈癌+子宫颈癌)+R=(宫颈癌+子宫颈癌))*(K=(危险度+危险因素+危险性因素+风险因素+相关因素+保护因素+影响因素+高危因素+协同因素+预期+预测)+T=(危险度+危险因素+危险性因素+风险因素+相关因素+保护因素+影响因素+高危因素+协同因素+预期+预测)))-T=(预后+复发+转移)	240

QVIP; year 2014 to 6 July 2016. Search run on 6 July 2016.

#	Search terms	Hits
1	(主题:(宫颈癌+子宫颈癌)*题名或关键词:(危险度+危险因素+危险性因素+风险因素+相关因素+保护因素+影响因素+高危因素+协同因素+预期+预测))^题名:(预后+复发+转移)	248

Appendix Figure 1. Flow chart of the systematic review and the literature update

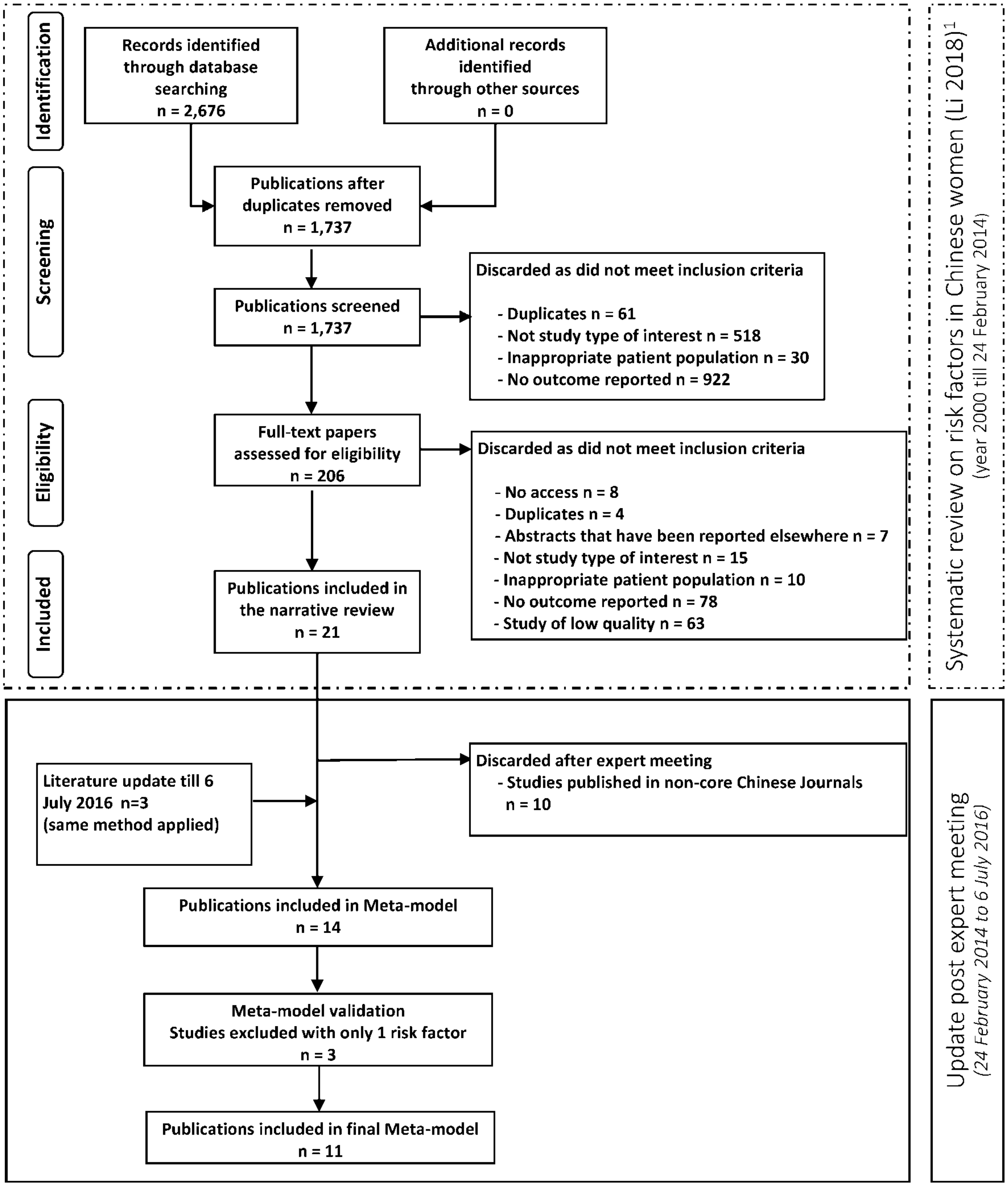

Appendix Figure 2. Overview of the risk factors from previously published systematic review[1] and the literature update

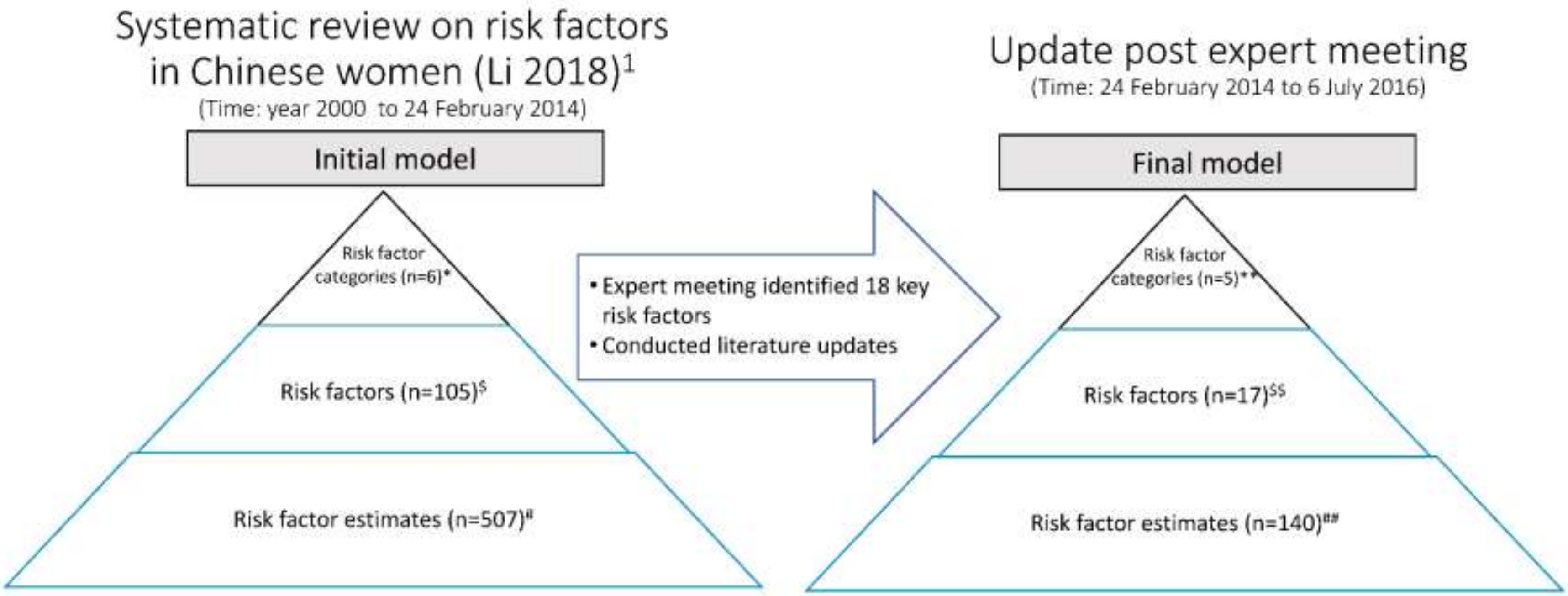

* Six categories are: socio-demographics, life style, sexual behavior and marital status, gestational risk factors, CC screening and gynecological disease, other factors. $ Each risk factor may correspond to different questions in different studies (i.e. education, below or above high school, below or above primary school, high school, college or university). # Individual entries of each risk factor by each individual question. ** Category "other factors" is removed. $$ Experts selected 18 risk factor groups, however, after the update post expert meeting, no studies were identified for one of the selected risk factor group, so only the remaining 17 risk factor groups were included in the final model. The missing risk factor is 'history of cervical treatment'. ## All risk estimates are listed in Appendix Tables 3-16.

Appendix 2. Selection criteria for the systematic literature review

Appendix Table 1. Selection criteria for the systematic literature review

Criteria	Inclusion criteria	Exclusion criteria
Population	Adult or adolescent Chinese women in Mainland China	Not focusing on humans<br>Not focusing on adult or adolescent females<br>Not focusing on Chinese (Mainland China)
Intervention	No restriction on interventions	No restriction on interventions
Comparators	No restriction on comparators	No restriction on comparators
Outcome	Risk factors for cervical cancer with numerical values<br>Risk factors chosen by the experts[a]	Not including outcome of interest (e.g., no numerical values reported)<br>Risk factors not chosen by the experts[a]
Study types/characteristics	Observational studies (e.g., case-control, cohort studies) that could be used to identify risk factors	Not study type of interest (e.g., letter, commentary, economic evaluation, clinical trials)
	Sample size N≥20	Observational studies with a very small sample size (i.e., N<20)
	Published between March 2014 and 6th July 2016	Published before March 2014[b]
	Quality of the study - studies must be published in core-journals listed in the PKU, CSCD or CSSCI indexes (applies only to non-English journals)	Non-core journals
	Duplicates	Duplicate of an existing entry

	Abstract that is not reported elsewhere	The abstract and the content were reported in another publication. This exclusion criterion was only applied if the numerical values were the same as in the full publication. In case of multiple abstracts reporting the same numerical values, only the abstract of the full publication was considered.

[a]Applied only for full-text screening

[b]Articles before 2014 were identified in the initial systematic literature review[1]

CSCD, China Science Citation Database; CSSCI, Chinese Social Sciences Citation Index, PKU, Peking University Chinese Core Journal List

Appendix 3. Process of expert meeting to identify the most important risk factors to include in the final meta-model

Appendix Table 2. Process of expert meeting to identify the most important risk factors to include in the final meta-model

Step	Tasks undertaken
1	Introduction of attending experts, study background and study objectives, and objectives of expert meeting
2	Summary of systematic literature review<br>Initial selection of risk factors and statistical methods
3	Presentation of risk factor rankings by frequency reported, odds ratio and prevalence
4	Discussion of the relevance and plausibility of risk factors based on frequency reported, odds ratio and prevalence
5	Discussion and validation of statistical methods<br>Comments on how to validate the model and potential improvements
6	Selection of the most important risk factors, ideally aiming for 10–15 risk factors
7	Suggestions on potential applications of the meta-model

Appendix 4. Prevalence search

A search was conducted to obtain population-based prevalence estimates for each of the 18 risk factors chosen by the experts. Data sources with population-based estimates were given the highest priority in order to minimize the bias associated with the prevalence estimate for each risk factor. A tiered system was used for differentiation of the data sources.

First, a targeted search in public (health) databases providing Chinese population based estimates was performed (Tier 1). This tier of data sources was considered the most relevant because the provided prevalence estimates most often rely on Chinese census data or official surveys. If estimates were available in both Chinese and English databases, the Chinese database estimates were preferred.

If no results were obtained by searching the first tier of data, the search next focused on prevalence estimates of cervical cancer risk factors reported in large cohort studies ($n \geq 1000$) and public surveys (Tier 2). A targeted search was performed using the databases Wanfang, CNKI and QCVIP, English and Chinese search engines such as Google (www.google.cn or www.google.com), Google Scholar (www.google.scholar.cn), Baidu (www.baidu.com), and Baiduxueshu (http://xueshu.baidu.com) (Chinese Google Scholar), and Chinese public surveys such as the Chinese Health and Nutrition survey (http://www.cpc.unc.edu/projects/china/data). If multiple estimates were obtained in this tier, priority was given to public surveys, followed by cohort studies with a large size or including multiple centers.

If no results were obtained by searching large cohort studies and public surveys, grey literature was searched using the same search engines (Tier 3). Grey literature publications may include, but are not limited to: reports such as pre-prints, preliminary and advanced reports, state of the art reports, statistical reports or technical reports; fact sheets; theses; etc. Publishers are considered non-commercial, such as non-governmental organizations. If estimates were obtained from grey literature, the quality, validity and applicability of these estimates were discussed between both reviewers.

If no results were obtained from the first three tiers, the prevalence estimate of the CC risk factor in Chinese women was considered equal to the prevalence of the control group in the original case-control study from which the risk factor and the risk factor estimates had been derived (Tier 4). If risk factors were reported in multiple studies and no judgement could be made about which prevalence estimate was considered to be most representative, the average across studies was used.

If no results were available from the underlying case control study, e.g. due to lack of data about the prevalence in the control group, the search then focused on non-Chinese data sources (Tier 5), using the same approach as the first three tiers. Data sources were prioritized based on geographical and cultural proximity to China (e.g. data sources from the Asian region were preferred over European region estimates). If estimates were obtained from non-Chinese data sources, the quality, validity and applicability of these estimates were discussed between both reviewers.

Appendix 5. Random-effects meta-analysis

The total variance (Q-statistic) was calculated as follows:[2]

$$Q_i = \sum_{ik} w_{ik} \; (\log(R_{ik}) - \frac{\sigma_{ik}^2}{2} - \overline{M_\iota})^2$$

where R_{ik} is the mean risk for the patient *i* based on study *k*, and σ_{ik}^2 is the associated variance. The expression $\log(R_{ik}) - \frac{\sigma_{ik}^2}{2}$ represents the mean of the log-risk for patient *i* in study *k*.[3] $\overline{M_\iota}$: precision-weighted average of the mean log-risks for patient *i* using the inverse of the within-study variances w_k; w_k: inverse of the within-study variance, i.e. $w_{ik} = \frac{1}{{\sigma_{ik}}^2}$.

The degrees of freedom (*df*) = Number of studies -1 and thus the between study variance τ_i^2 can be calculated as:

$$\tau_i^2 = \begin{cases} \frac{Q_i - df}{C_i}, if \; Q_i > df \\ 0, if \; Q_i \; \leq df \end{cases}$$

where C_i: scaling factor to scale the weighted sum of squares Q (i.e. total variance) with

$$C_i = \sum_{ik} w_{ik} - \frac{\sum_{ik} w_{ik}^2}{\sum_{ik} w_{ik}}$$

Aggregating the log-study risks $\log(R_{ik})'$ in the random-effects meta-analysis, each log-risk was then weighted by an inverse-variance weighted method using weight w_{ik}^* with

$$w_{ik}^* = \frac{1}{v_{ik}^*}$$

where $v_{ik}^* = \sigma_{i,k}^2 + \tau_i^2$.

Thus, the expected log-risk is:

$$E[\log(R_i)] = \frac{\sum_k w_{ik}^* \ \log(R_{ik})'}{\sum_k w_{ik}^*}$$

and the variance of the overall estimated log-risk is:

$$v_i^* = \frac{1}{\sum_k w_{ik}^*}.$$

Using a back transformation, the overall risk R_i is obtained by:

$$R_i = \exp\left(E[\log(R_i)] + \frac{v_i^*}{2}\right)$$

and the corresponding variance by:

$$\mathrm{Var}_i = (\exp(v_i^*) - 1)\exp(2E[\log(R_i)] + v_i^*)$$

The corresponding lower bound (LB) and upper bound (UB) of the 95% CI were calculated as follows:

$$LB_i = \exp\left(E[\log(R_i)] + \frac{v_i^*}{2} - 1.96\sqrt{v_i^*}\right)$$

$$UB_i = \exp\left(E[\log(R_i)] + \frac{v_i^*}{2} + 1.96\sqrt{v_i^*}\right).$$

Appendix 6. Ranking of initial risk factors

Appendix Figure 3. Top 30 risk factors ranked by number of studies in which risk factor was reported [§]

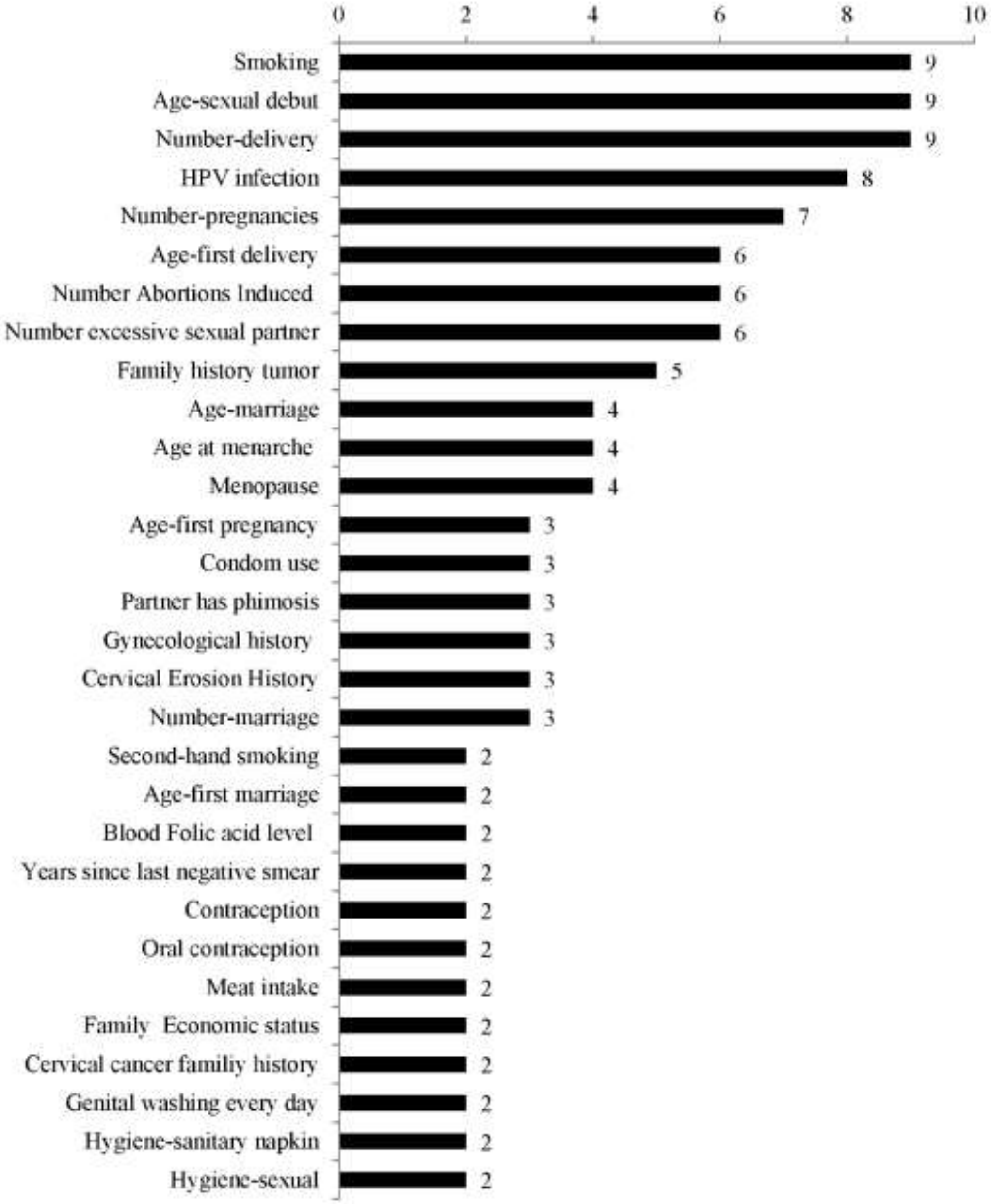

HPV, human papillomavirus; [§] risk factor definitions see Appendix Table 3

Appendix Figure 4. Top 30 risk factors ranked by prevalence (in %) §

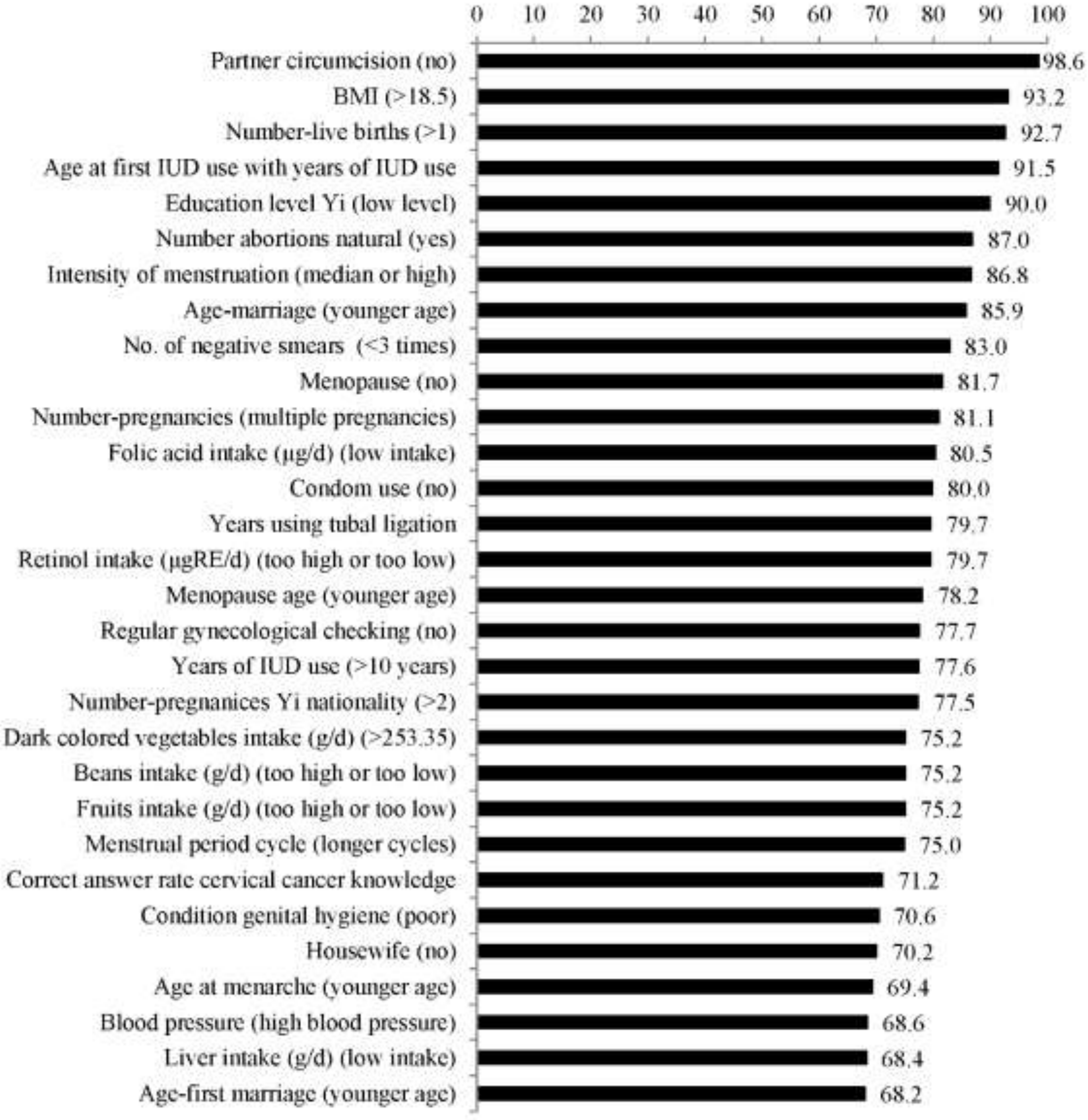

BMI, body mass index; IUD, intrauterine device; § risk factor definitions see Appendix Table 3

Appendix Figure 5. Lowest 30 risk factors ranked by prevalence (in%) §

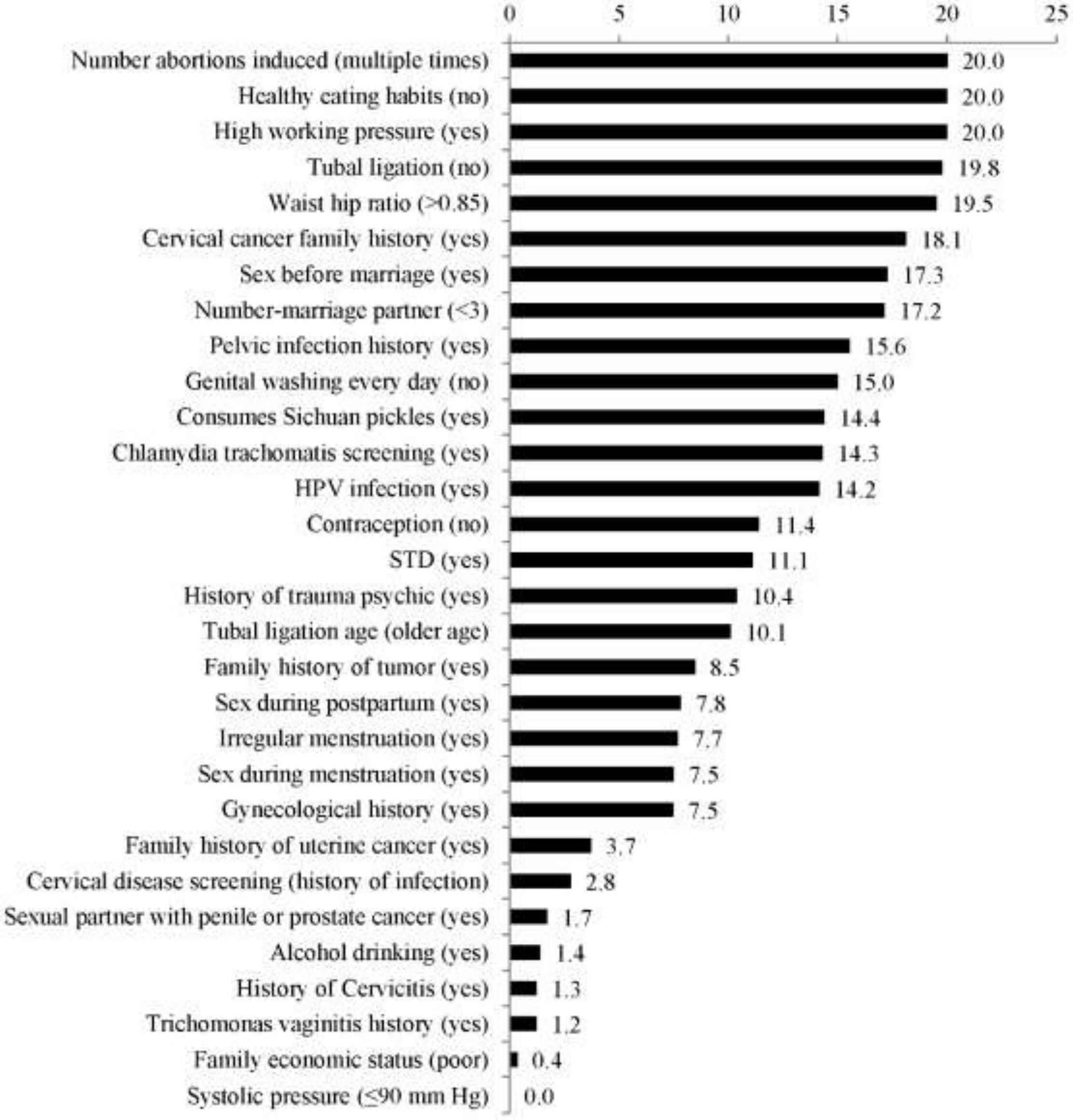

HPV, human papillomavirus; STD, sexually transmitted disease; § risk factor definitions see Appendix Table 3

Appendix Table 3. Definition of the 105 risk factors

Name risk factor	Risk factor description	Match with risk factors identified in SLR	Risk factor category identified in SLR
Intellectual Job (no)	Yes/No; yes is reference	Occupation	Socio-demographic
Family Economic status (poor)	Wealthy/poor; wealthy is reference	Economic status	Socio-demographic
Education level school (secondary school or below)	Below high school/High school and above; high school and above is reference	Education	Socio-demographic
Housewife (no)	Yes/No; yes is reference	Occupation	Socio-demographic
Income (low)	Various level; high is reference	Economic status	Socio-demographic
Worker (yes)	Yes/No; no is reference	Occupation	Socio-demographic
Education level all (lower)	Primary school level or below/Secondary school/2 yearcollege degree or above; 2 year college degree or above is reference	Education	Socio-demographic
Education level Yi (low level)	High/Low; high is reference	Education	Socio-demographic
Bleeding during sex (yes)	Yes/No; no is reference	Bleeding during sex	Sexual behavior
Duration-marriage (≥4 years)	Various years; <4 is reference	Duration-marriage	Sexual behavior
Sex before marriage (yes)	Yes/No; no is reference	Sex before marriage	Sexual behavior
Number-sexual partner of partner (>1)	Various numbers; the more the higher risk	Number-sexual partner of partner	Sexual behavior
Number excessive sexual partner (≥1)	Various numbers; the more the higher risk	Number-sexual partner	Sexual behavior
Age-sexual debut (younger age)	Various age; the younger the higher risk	Age-sexual debut	Sexual behavior
Number-marriage (>1)	Marriage numbers; the more the higher risk	Number-marriage	Sexual behavior
Age-marriage (younger age)	Various age; the younger the higher risk	Age-marriage	Sexual behavior
Age-first marriage (younger age)	Various age; the younger the higher risk	Age-marriage	Sexual behavior
Sex during menstruation (yes)	Yes/No; no is reference	Time-sex since delivery/during cycles	Sexual behavior
Duration sex-partner (yes)	Yes/No; no is reference	Duration-marriage	Sexual behavior
Sex during postpartum (yes)	Yes/No; no is reference	Time-sex since delivery/during cycles	Sexual behavior
Status-marriage (unmarried/divorced/remarried)	Various status; married is reference	Status-marriage	Sexual behavior
Number-marriage partner (<3)	Various number of marriages; the less the higher risk	Number-marriage partner	Sexual behavior

Life-time sexual partners numbers (>1)	Various numbers; the more the higher risk	Number-sexual partner	Sexual behavior
HPV infection (yes)	Yes/No; no is reference	HPV infection	Screening and disease
History of Cervicitis (yes)	Yes/No; no is reference	Gynecological disorder	Screening and disease
Cervical disease screening (history of infection)	No vagina or cervix disease/History of vaginal infection/cervicitis/Never examined; No vagina/cervix disease is reference	Cervical screening history	Screening and disease
STD (yes)	Yes/No; no is reference	STD	Screening and disease
Cervical cancer family history (yes)	Yes/No; no is reference	Gynecological disorder	Screening and disease
Years since last negative smear (>2 years)	Various years; 0-2 is reference	Cervical screening history	Screening and disease
High working pressure (yes)	Yes/No; no is reference	Mental health	Screening and disease
Family history uterine cancer (yes)	Yes/No; no is reference	Gynecological disorder	Screening and disease
Cervical Erosion History (yes)	Yes/No; no is reference	Gynecological disorder	Screening and disease
Gynecological checking location (self)	Hospital/self, hospital is reference	Cervical screening history	Screening and disease
Gynecological history (yes)	Yes/No; no is reference	Gynecological disorder	Screening and disease
No. negative smears (<3 times)	Number of smears; >3 is reference	Cervical screening history	Screening and disease
Pelvic Infection History (yes)	Yes/No; no is reference	Gynecological disorder	Screening and disease
Trichomonas vaginitis history (yes)	Yes/No; no is reference	Gynecological disorder	Screening and disease
History of trauma psychic (yes)	Yes/No; no is reference	Mental health	Screening and disease
Family history tumor (yes)	Yes/No; no is reference	Gynecological disorder	Screening and disease
Screened for HSV-2 (yes)	Yes/No; no is reference	Cervical screening history	Screening and disease
Chlamydia trachomatis Screening (yes)	Yes/No; no is reference	Cervical screening history	Screening and disease
Regular gynecological checking (no)	Yes/No; yes is reference	Cervical screening history	Screening and disease
Knowledge on own cervical condition (no)	Yes/No; yes is reference	Knowledge/awareness	Other factors
Systolic pressure (≤90mm Hg)	Various level; >90mm Hg is reference	Other bodily measure	Other factors
Waist hip ratio (>0.85)	Various level; <0.85 is reference	Other bodily measure	Other factors
Blood pressure (high blood pressure)	Various level; the higher the higher risk	Other bodily measure	Other factors

Blood Folic acid level (nmol/L)(low intake)	Various level; 15.76-19.92 is reference	Biomarker level	Other factors
Partner has phimosis (yes)	Yes/No; no is reference	Foreskin	Other factors
Partner circumcision (no)	Yes/No; yes is reference	Foreskin	Other factors
BMI (>18.5)	Various level; the higher the higher risk	Other bodily measure	Other factors
Folic acid intake (μg/d) (low intake)	Various intake; the lower the higher risk	Biomarker level	Other factors
Correct answer rate cervical cancer knowledge (low)	Various correction rate; the lower the higher risk	Knowledge/awareness	Other factors
Retinol intake (μgRE/d) (low intake)	Various level; 170.10-311.15 is reference	Biomarker level	Other factors
Sexual partner with penile/prostate cancer (yes)	Yes/No; no is reference	Penile disease	Other factors
Spouse has penile cancer (yes)	Yes/No; no is reference	Penile disease	Other factors
Condition Genital hygiene (poor)	Various level; the poorer the higher risk	Hygiene-genital	Life style
Genital washing every day (no)	Yes/No; yes is reference	Hygiene-genital	Life style
Personal health habits (≤ 4 times a week)	Various times of changing and washing (underwears/week; more than 4 times a week is reference)	Hygiene-general	Life style
Bathing facilities Access (no)	Yes/No; yes is reference	Hygiene-general	Life style
Personal hygiene (Poor or good)	Various level; fair is reference	Hygiene-general	Life style
Second hand smoking (yes)	Yes/No; no is reference	Addictions	Life style
Drinking tea often (no)	Yes/No; yes is reference	Water/beverage	Life style
Menstrual hygiene Score (>1)	Various score; the higher score the higher risk; scored based on poor hygiene during menstrual cycle, no genital washing, labor work and sexual intercourse during menstrual cycle, and no sanitary napkin	Hygiene-genital	Life style
Hygiene-sexual (no)	Washing during intercourse; Yes/No; yes is reference	Hygiene-sexual	Life style
Smoking (yes)	Yes/No; no is reference	Addictions	Life style
Dark colored vegetable intake (g/d) (<253.35)	Various intake; the lower the higher risk	Dietary intake	Life style
Vegetable intake (times/per week) (<7 times)	Various times a week; the fewer times the higher risk	Dietary intake	Life style
Healthy Eating habits (no)	Yes/No; yes is reference	Dietary intake	Life style

Beans intake (g/d) (too high or too low)	Various intake; 31.92-56.51 is reference	Dietary intake	Life style
Hygiene-sanitary napkin (no)	Yes/No; yes is reference	Hygiene-sanitary napkin	Life style
Alcohol drinking (yes)	Yes/No; no is reference	Water/beverage	Life style
Fruits intake (g/d) (too high or too low)	Various intake; 98.63-207.94 is reference	Dietary intake	Life style
Consume Sichuan pickles (yes)	Yes/No; no is reference	Dietary intake	Life style
Liver intake (g/d) (low intake)	Various intake; the lower the higher risk	Dietary intake	Life style
Meat intake (too high or too low)	Regular/normal meat consumption (2-6 times per day; 22.19 to 46.3 grams per day) is reference	Dietary intake	Life style
Number-live births (>1)	Live birth numbers; the more the higher risk	Number-live births	Gestational factors
Contraception other methods (yes)	Yes/No; no is reference	Contraception	Gestational factors
Number-pregnancies Yi (>2)	Various pregnancies; the more the higher risk	Number-pregnancies	Gestational factors
Irregular menstruation (yes)	Yes/No; no is reference	Menstruation-last,age	Gestational factors
Age-first pregnancy (older age)	Various age; the older the higher risk	Age-first pregnancy	Gestational factors
Number-delivery Yi (more)	Various deliveries; the more the higher risk	Number-delivery	Gestational factors
Number Abortions Induced (multiple times)	Various times; the more times the higher risk	History of non-full term live birth/termination	Gestational factors
Oral contraception (no)	Yes/No; yes is reference	Contraception	Gestational factors
Still birth (yes)	Yes/No; no is reference	History of non-full term live birth/termination	Gestational factors
Age at menarche (younger age)	Various age; the younger the higher risk	Menarche	Gestational factors
Number-delivery (>2)	Various deliveries; the more the higher risk	Number-delivery	Gestational factors
Duration of menstruation (>3)	Various days; the longer the higher risk	Menstruation-interval	Gestational factors
Intensity menstruation (median or high)	Various level; the higher intensity the higher risk	Menstruation-last,age	Gestational factors
Contraception (no)	Yes/No; yes is reference	Contraception	Gestational factors
Age-first delivery (younger age)	Various age; the younger the higher risk	Age-first delivery	Gestational factors
Number-pregnancies (multiple pregnancies)	Various pregnancies; the more the higher risk	Number-pregnancies	Gestational factors
Menopause (no)	Yes/No; yes is reference	Menstruation-last,age	Gestational factors

Years of IUD use (>10 years)	Various years; 1-10 years is reference	Contraception	Gestational factors
IUD age first use (>33)	Various age; the older the higher risk	Contraception	Gestational factors
Contraceptive ring (no)	Yes/No; yes is reference	Contraception	Gestational factors
Age at first IUD use with years of IUD use (33+)	Various age; the older the higher risk	Contraception	Gestational factors
Menstrual period cycle (longer cycles)	Various days; the longer the higher risk	Menstruation-interval	Gestational factors
Age at first tubal ligation with years since sterilization (33+)	Various age; the older the higher risk	Contraception	Gestational factors
Years using Tubal ligation (≥11)	Various years; 1-10 is reference	Contraception	Gestational factors
Menopause age (younger age)	Various ages; the younger the higher risk	Menstruation-last,age	Gestational factors
Condom use (no)	Yes/No; yes is reference	Contraception	Gestational factors
IUD use (no)	Yes/No; yes is reference	Contraception	Gestational factors
Tubal ligation (no)	Yes/No; no is reference	Contraception	Gestational factors
Number Abortions Natural (yes)	Yes/No; no is reference	History of non-full term live birth/termination	Gestational factors
Tubal ligation age (older age)	Various age; the older the higher risk	Contraception	Gestational factors

BMI, body mass index; HPV, human papillomavirus; HSV, herpes simplex virus; IUD, intrauterine device; SLR, systematic literature review; STD, sexually transmitted disease

Appendix 7. Results for risk factors included in final meta-model

Appendix Table 4. Educational level

Study reference	Risk factors	Variables	OR	95% CI of OR (LB,UB)	Population prevalence (%)	Adjusted based on	Note
Kan et al,2009[4]	Educational level	Below high school	1	(1,1)	76.6	NA	U
Kan et al,2009[4]	Educational level	High school or above	0.846	(0.652,0.977)	23.4	NA	U
Ye et al,2014[5]	Educational level	Below high school	1.467	(1.408,1.478)	76.6	NA	M
Ye et al,2014[5]	Educational level	High school or above	1	(1,1)	23.4	NA	M
Hu et al,2014[6]	Educational level	College and above	1	(1,1)	10.44	NA	M
Hu et al,2014[6]	Educational level	High school	2.034	(0.668,2.643)	12.97	NA	M
Hu et al,2014[6]	Educational level	Junior high school	1.531	(0.668,2.643)	39.39	NA	M
Hu et al,2014[6]	Educational level	Primary school	1.765	(0.668,2.643)	27.46	NA	M
Hu et al,2014[6]	Educational level	Below primary school	2.034	(0.668,2.643)	9.74	NA	M

CI, confidence interval; LB, lower bound; M, Multivariate analysis; NA, Not applicable; OR, odds ratio; U, Univariate analysis; UB, Upper bound

Appendix Table 5. Occupations

Study reference	Risk factors	Variables	OR	95% CI of OR (LB,UB)	Population prevalence (%)	Adjusted based on	Note
Kan et al,2009[4]	Intellectual job	No	1	(1,1)	87.67	NA	U
Kan et al,2009[4]	Intellectual job	Yes	0.268	(0.117,0.403)	12.33	NA	U

CI, confidence interval; LB, lower bound; NA, Not applicable; OR, odds ratio; U, Univariate analysis; UB, Upper bound

Appendix Table 6. Smoking and second-hand smoking

Study reference	Risk factors	Variables	OR	95% CI of OR (LB,UB)	Population prevalence (%)	Adjusted based on	Note
Peng et al,1991[7]	Cigarette smoking	No	1	(1,1)	97.7	Age + other factor (adjusted for age, income, residence, HPV 16/33, age at first marriage)	M
Peng et al,1991[7]	Cigarette smoking	Yes	1.2	(0.5,2.8)	2.3	Age + other factor (adjusted for age, income, residence, HPV 16/33, age at first marriage)	M
Dong et al,1998[8]	Cigarette smoking	No	1	(1,1)	97.7	Adjusted for age, HPV infection status	M
Dong et al,1998[8]	Cigarette smoking	Yes	2	(0.66,6.2)	2.3	Adjusted for age, HPV infection status	M
Li et al,2000[9]	Smoking index (Smoking index = cigarette/day × number of smoking year)	0	1	(1,1)	97.7	Adjusted for age, IUD use, tubal ligation, number of induced and spontaneous abortions, age at first birth, number of deliveries, months of breast feeding	M
Li et al,2000[9]	Smoking index (Smoking index = cigarette/day × number of smoking year)	> 200	1.76	(1.05,2.96)	0.5	Adjusted for age, IUD use, tubal ligation, number of induced and spontaneous abortions, age at first birth, number of deliveries, months of breast feeding	M
Li et al,2000[9]	Smoking index (Smoking index = cigarette/day × number of smoking year)	≤ 200	1.61	(1.02,2.54)	1.8	Adjusted for age, IUD use, tubal ligation, number of induced and spontaneous abortions, age at first birth, number of deliveries, months of breast feeding	M
Kan et al,2009[4]	Smoking	No	1	(1,1)	97.7	NA	M
Kan et al,2009[4]	Smoking	Yes	2.946	(1.758,6.281)	2.3	NA	M
Li et al,2011[10]	Second-hand smoking	No	1	(1,1)	51.17	NA	M

Li et al,2011[10]	Second-hand smoking	Yes	1.844	(1.038,3.278)	48.83	NA	M

CI, confidence interval; HPV, human papillomavirus; IUD, intra-uterine device; LB, lower bound; M, Multivariate analysis; NA, Not applicable; OR, odds ratio; U, Univariate analysis; UB, Upper bound

Appendix Table 7. Poor sexual hygiene (washing or not before sex)

Study reference	Risk factors	Variables	OR	95% CI of OR (LB,UB)	Population prevalence (%)	Adjusted based on	Note
Li et al,2011[10]	Pay attention to hygiene at sexual intercourses	No	1	(1,1)	17.94	NA	M
Li et al,2011[10]	Pay attention to hygiene at sexual intercourses	Yes	0.329	(0.182,0.595)	82.06	NA	M

CI, confidence interval; LB, lower bound; M, Multivariate analysis; NA, Not applicable; OR, odds ratio; UB, Upper bound

Appendix Table 8. Age at sexual debut

Study reference	Risk factors	Variables	OR	95% CI of OR (LB,UB)	Population prevalence (%)	Adjusted based on	Note
Zhang et al,1989a[11]	Age at first sexual intercourse	≤16	1	(1,1)	2.03	NA	U
Zhang et al,1989a[11]	Age at first sexual intercourse	≥ 21	0.35	(0.12,0.94)	74.87	NA	U
Zhang et al,1989a[11]	Age at first sexual intercourse	17-18	0.78	(0.45,1.36)	6.83	NA	U
Zhang et al,1989a[11]	Age at first sexual intercourse	19-20	0.74	(0.41,1.35)	16.27	NA	U
Cai et al,2008[12]	Age at first sexual intercourse	< 18	3.71	(1.82,16.81)	4.53	NA	M
Cai et al,2008[12]	Age at first sexual intercourse	> 23	1	(1,1)	38.72	NA	M
Cai et al,2008[12]	Age at first sexual intercourse	18-20	1.46	(0.31,6.79)	20.60	NA	M
Cai et al,2008[12]	Age at first sexual intercourse	21-23	0.41	(0.08,2.2)	36.15	NA	M
Wang et al,2004[13]	Age at first sexual intercourse	> 20 years	0.499	(0.305,0.818)	74.87	NA	M

Wang et al,2004[13]	Age at first sexual intercourse	≤ 20 years	1	(1,1)	25.13	NA	M
Kan et al,2009[4]	Age at first sexual intercourse	> 20	1	(1,1)	74.87	NA	M
Kan et al,2009[4]	Age at first sexual intercourse	≤ 20	4.529	(2.187,9.017)	25.13	NA	M
Ye et al,2014[5]	Age at first sexual intercourse	< ** years old	1.421	(1.142,2.163)		NA	M
Ye et al,2014[5]	Age at first sexual intercourse	> ** years old	1	(1,1)		NA	M

CI, confidence interval; LB, lower bound; M, Multivariate analysis; NA, Not applicable; OR, odds ratio; U, Univariate analysis; UB, Upper bound

Appendix Table 9. Number of sexual partners

**Study reference**	**Risk factors**	**Variables**	**OR**	**95% CI of OR (LB,UB)**	**Population prevalence (%)**	**Adjusted based on**	**Note**
Zhang et al,1989a[11]	No. of non-marital sexual partners	1	2.47	(0.82,7.45)	10.8	Adjust for screening history	M
Zhang et al,1989a[11]	No. of non-marital sexual partners	≥ 2	3.87	(1.56,9.55)	4.8	Adjust for screening history	M
Zhang et al,1989a[11]	No. of non-marital sexual partners	0	1	(1,1)	84.4	Adjust for screening history	M
Cai et al,2008[12]	Lifetime no. of sexual partners	≥ 2	1.01	(0.32,3.14)	29.6	NA	M
Cai et al,2008[12]	Lifetime no. of sexual partners	0-1	1	(1,1)	70.4	NA	M
Kan et al,2009[4]	No. of sexual partners	< 3	1	(1,1)	70.4	NA	M
Kan et al,2009[4]	No. of sexual partners	≥ 3	4.012	(1.979,7.846)	29.6	NA	M

CI, confidence interval; LB, lower bound; M, Multivariate analysis; NA, Not applicable; OR, odds ratio; UB, Upper bound

Appendix Table 10. Age at first delivery

Study reference	Risk factors	Variables	OR	95% CI of OR (LB,UB)	Population prevalence (%)	Adjusted based on	Note
Zhang et al,1989a[11]	Age at first delivery	≤19	1	(1,1)	3.46	NA	U
Zhang et al,1989a[11]	Age at first delivery	≥ 22	0.6	(0.34,1.05)	83.17	NA	U
Zhang et al,1989a[11]	Age at first delivery	20-21	0.86	(0.51,1.46)	13.36	NA	U
Wang et al,2004[13]	Age at first delivery	≤ 20	14.24	(6.89,29.41)	8.72	NA	U
Wang et al,2004[13]	Age at first delivery	≥ 25	1	(1,1)	51.26	NA	U
Wang et al,2004[13]	Age at first delivery	21-24	4.41	(2.44,7.98)	40.02	NA	U
Kan et al,2009[4]	Age at first delivery	≤ 21	1.295	(1.022,2.073)	16.83	NA	U
Kan et al,2009[4]	Age at first delivery	> 21	1	(1,1)	83.17	NA	U
Hu et al,2014[6]	Age at first delivery	15	0.827	(0.679,1.007)	0.011	NA	M
Hu et al,2014[6]	Age at first delivery	16	0.817	(0.662,1.007)	0.098	NA	M
Hu et al,2014[6]	Age at first delivery	17	0.806	(0.645,1.008)	0.378	NA	M
Hu et al,2014[6]	Age at first delivery	18	0.796	(0.629,1.008)	0.995	NA	M
Hu et al,2014[6]	Age at first delivery	19	0.786	(0.613,1.009)	1.981	NA	M
Hu et al,2014[6]	Age at first delivery	20	0.776	(0.597,1.009)	5.253	NA	M
Hu et al,2014[6]	Age at first delivery	21	0.766	(0.582,1.01)	8.110	NA	M
Hu et al,2014[6]	Age at first delivery	22	0.757	(0.567,1.01)	9.705	NA	M
Hu et al,2014[6]	Age at first delivery	23	0.747	(0.552,1.011)	11.705	NA	M
Hu et al,2014[6]	Age at first delivery	24	0.738	(0.538,1.011)	10.500	NA	M
Hu et al,2014[6]	Age at first delivery	25	0.729	(0.525,1.012)	8.241	NA	M

Hu et al,2014[6]	Age at first delivery	26	0.719	(0.511,1.012)	7.309	NA	M
Hu et al,2014[6]	Age at first delivery	27	0.710	(0.498,1.013)	6.502	NA	M
Hu et al,2014[6]	Age at first delivery	28	0.701	(0.486,1.013)	6.478	NA	M
Hu et al,2014[6]	Age at first delivery	29	0.693	(0.473,1.014)	4.520	NA	M
Hu et al,2014[6]	Age at first delivery	30	0.684	(0.461,1.014)	3.330	NA	M
Hu et al,2014[6]	Age at first delivery	31	0.675	(0.449,1.015)	2.619	NA	M
Hu et al,2014[6]	Age at first delivery	32	0.667	(0.438,1.015)	1.996	NA	M
Hu et al,2014[6]	Age at first delivery	33	0.658	(0.427,1.015)	1.426	NA	M
Hu et al,2014[6]	Age at first delivery	34	0.650	(0.416,1.016)	1.262	NA	M
Hu et al,2014[6]	Age at first delivery	35	0.642	(0.405,1.016)	1.083	NA	M
Hu et al,2014[6]	Age at first delivery	36	0.634	(0.395,1.017)	0.945	NA	M
Hu et al,2014[6]	Age at first delivery	37	0.626	(0.385,1.017)	0.821	NA	M
Hu et al,2014[6]	Age at first delivery	38	0.618	(0.375,1.018)	0.719	NA	M
Hu et al,2014[6]	Age at first delivery	39	0.610	(0.366,1.018)	0.611	NA	M
Hu et al,2014[6]	Age at first delivery	≥ 40	0.603	(0.356,1.019)	3.402	NA	M

CI, confidence interval; LB, lower bound; M, Multivariate analysis; NA, Not applicable; OR, odds ratio; U, Univariate analysis; UB, Upper bound

Appendix Table 11. Age at first pregnancy

**Study reference**	**Risk factors**	**Variables**	**OR**	**95% CI of OR (LB,UB)**	**Population prevalence (%)**	**Adjusted based on**	**Note**
Wang et al,2004[13]	Age at first pregnancy	≤ 20	13.71	(6.77,27.77)	12.22	NA	U
Wang et al,2004[13]	Age at first pregnancy	≥ 25	1	(1,1)	44.44	NA	U

Wang et al,2004[13]	Age at first pregnancy	21-24	3.23	(1.76,5.94)	43.34	NA	U
Kan et al,2009[4]	Age at first pregnancy	> 21	1	(1,1)	87.78	NA	U
Kan et al,2009[4]	Age at first pregnancy	≤ 21	2.283	(1.515,3.816)	12.22	NA	U

CI, confidence interval; LB, lower bound; NA, Not applicable; OR, odds ratio; U, Univariate analysis; UB, Upper bound

Appendix Table 12. Number of deliveries

**Study reference**	**Risk factors**	**Variables**	**OR**	**95% CI of OR (LB,UB)**	**Population prevalence (%)**	**Adjusted based on**	**Note**
Zhang et al,1989a[11]	No. of normal deliveries	≥ 3	1.89	(0.69,5.6)	13.4	NA	U
Zhang et al,1989b[14]	No. of normal deliveries	1-2	2.57	(0.88,8.06)	61.85	NA	U
Zhang et al,1989b[14]	No. of normal deliveries	0	1	(1,1)	24.77	NA	U
Peng et al,1991[7]	No. of deliveries	≤ 2	1	(1,1)	86.62	Age + other factor (adjusted for age, income, residence, HPV 16/33, age at first marriage, cigarette smoking)	M
Peng et al,1991[7]	No. of deliveries	≥ 5	1.5	(0.6,3.6)	1.05	Age + other factor (adjusted for age, income, residence, HPV 16/33, age at first marriage, cigarette smoking)	M
Peng et al,1991[7]	No. of deliveries	3-4	1.1	(0.4,2.9)	12.34	Age + other factor (adjusted for age, income, residence, HPV 16/33, age at first marriage, cigarette smoking)	M
Dong et al,1998[8]	No. of deliveries	> 3	1.5	(0.24,9.3)	3.96	Adjusted for age, HPV infection status	M
Dong et al,1998[8]	No. of deliveries	1-2	1.2	(0.52,6.7)	61.85	Adjusted for age, HPV infection status	M

Dong et al,1998[8]	No. of deliveries	0	1	(1,1)	24.77	Adjusted for age, HPV infection status	M
Wang et al,2004[13]	No. of deliveries	≤ 2	1	(1,1)	86.62	NA	M
Wang et al,2004[13]	No. of deliveries	3-4	2.293	(1.312,4.008)	12.34	NA	M
Kan et al,2009[4]	No. of deliveries	< 3	1	(1,1)	86.62	NA	U
Kan et al,2009[4]	No. of deliveries	≥ 3	2.795	(1.93,4.872)	13.38	NA	U
Li et al,2011[10]	No. of deliveries	> 2	2.267	(1.278,4.021)	13.38	NA	M
Li et al,2011[10]	No. of deliveries	≤ 2	1	(1,1)	86.62	NA	M
Ye et al,2014[5]	No. of deliveries	> **	1.201	(0.675,2.206)		NA	M
Ye et al,2014[5]	No. of deliveries	< **	1	(1,1)		NA	M

CI, confidence interval; HPV, human papillomavirus; LB, lower bound; M, Multivariate analysis; NA, Not applicable; OR, odds ratio; U, Univariate analysis; UB, Upper bound

Appendix Table 13. Number of pregnancies

**Study reference**	**Risk factors**	**Variables**	**OR**	**95% CI of OR (LB,UB)**	**Population prevalence (%)**	**Adjusted based on**	**Note**
Dong et al,1998[8]	No. of pregnancies	> 3	0.72	(0.13,4.1)	13.01	Adjusted for age, HPV infection status	M
Dong et al,1998[8]	No. of pregnancies	1-2	0.24	(0.043,1.3)	52.47	Adjusted for age, HPV infection status	M
Dong et al,1998[8]	No. of pregnancies	0	1	(1,1)	17.14	Adjusted for age, HPV infection status	M
Cai et al,2008[12]	No. of pregnancies	≥4	3.49	(0.61,20.1)	13.01	NA	M
Cai et al,2008[12]	No. of pregnancies	0-1	1	(1,1)	42.35	NA	M
Cai et al,2008[12]	No. of pregnancies	2-3	1.67	(0.47,5.92)	44.65	NA	M
Wang et al,2004[13]	No. of pregnancies	4	1.47	(0.75,2.87)	8.11	NA	U

Wang et al,2004[13]	No. of pregnancies	≤ 3	1	(1,1)	86.99	NA	U
Wang et al,2004[13]	No. of pregnancies	≥ 5	4.29	(1.96,8.89)	4.9	NA	U
Kan et al,2009[4]	No. of pregnancies	≥ 3	1.371	(1.089,2.147)	30.39	NA	U
Kan et al,2009[4]	No. of pregnancies	< 3	1	(1,1)	69.61	NA	U

CI, confidence interval; HPV, human papillomavirus; LB, lower bound; M, Multivariate analysis; NA, Not applicable; OR, odds ratio; U, Univariate analysis; UB, Upper bound

Appendix Table 14. Contraception methods

**Study reference**	**Risk factors**	**Variables**	**OR**	**95% CI of OR (LB,UB)**	**Population prevalence (%)**	**Adjusted based on**	**Note**
Li et al,2000[9]	IUD use	No	1	(1,1)	49.43	Adjusted for age, HPV infection status	M
Li et al,2000[9]	IUD use	Yes	0.89	(0.73,1.08)	50.57	Adjusted for age, HPV infection status	M
Li et al,2000[9]	No. of years of IUD use	> 10	0.68	(0.44,1.04)	26.26	Adjusted for age, HPV infection status	M
Li et al,2000[9]	No. of years of IUD use	1-10	0.63	(0.4,0.98)	24.31	Adjusted for age, HPV infection status	M
Li et al,2000[9]	No. of years of IUD use	0	1	(1,1)	49.43	Adjusted for age, HPV infection status	M
Kan et al,2009[4]	Using condom	No	1	(1,1)	89.66	NA	M
Kan et al,2009[4]	Using condom	Yes	0.612	(0.547,0.816)	10.34	NA	M

CI, confidence interval; HPV, human papillomavirus; IUD, intra-uterine device; LB, lower bound; M, Multivariate analysis; NA, Not applicable; OR, odds ratio; UB, Upper bound

Appendix Table 15. Menopause

Study reference	Risk factors	Variables	OR	95% CI of OR (LB,UB)	Population prevalence (%)	Adjusted based on	Note
Wang et al,2004[13]	Menopause	No	1	(1,1)	59.6	NA	M
Wang et al,2004[13]	Menopause	Yes	0.68	(0.465,0.996)	40.43	NA	M
Kan et al,2009[4]	Menopause	No	1	(1,1)	59.6	NA	M
Kan et al,2009[4]	Menopause	Yes	0.507	(0.394,0.715)	40.43	NA	M

CI, confidence interval; LB, lower bound; M, Multivariate analysis; NA, Not applicable; OR, odds ratio; UB, Upper bound

Appendix Table 16. HPV infection

Study reference	Risk factors	Variables	OR	95% CI of OR (LB,UB)	Population prevalence (%)	Adjusted based on	Note
Peng et al,1991[7]	HPV-16/33	No	1	(1,1)	94.943	Age + other factor (adjusted for age, income, residence, age at first marriage, and cigarette smoking)	M
Peng et al,1991[7]	HPV-16/33	Yes	32.9	(7.7,141.1)	5.057	Age + other factor (adjusted for age, income, residence, age at first marriage, and cigarette smoking)	M
Dong et al,1998[8]	HPV infection (16,31,33,58)	Yes	21	(9.3,49)	8.734	Adjusted for age	M
Dong et al,1998[8]	HPV infection (16,31,33,58)	No	1	(1,1)	82.515	Adjusted for age	M
Cai et al,2008[12]	HPV Positive (16/58)	No	1	(1,1)	82.515	NA	M
Cai et al,2008[12]	HPV Positive (16/58)	Yes	75.79	(23.22,247.41)	6.587	NA	M
Kan et al,2009[4]	HPV infection (16)	No	1	(1,1)	96.26	NA	M

Kan et al,2009[4]	HPV infection (16)	Yes	21.025	(14.996,29.476)	3.74	NA	M
Ye et al,2014[5]	HPV infection	Yes	12.412	(7.284,27.402)	14.891	NA	M
Ye et al,2014[5]	HPV infection	No	1	(1,1)	85.109	NA	M
Hu et al,2014[6]	High-risk HPV infection (16, 18/45, 52, 58)	No	1	(1,1)	90.117	NA	M
Hu et al,2014[6]	High-risk HPV infection (16, 18/45, 52, 58)	Yes	7.626	(2.126,27.383)	9.883	NA	M

CI, confidence interval; HPV, human papillomavirus; LB, lower bound; M, Multivariate analysis; NA, Not applicable; OR, odds ratio; UB, Upper bound

Appendix Table 17. Cervical/gynecological screening history

**Study reference**	**Risk factors**	**Variables**	**OR**	**95% CI of OR (LB,UB)**	**Population prevalence (%)**	**Adjusted based on**	**Note**
Zhang et al,1989b[14]	No. of negative smears	> 3	0.33	(0.12,0.76)	17	To observe one variable when the other variable was stratified	M
Zhang et al,1989b[14]	No. of negative smears	0-1	1	(1,1)	67.2	To observe one variable when the other variable was stratified	M
Zhang et al,1989b[14]	No. of negative smears	2-3	0.47	(0.2,1.01)	15.8	To observe one variable when the other variable was stratified	M
Zhang et al,1989b[14]	Years since last negative smear	≥ 8	11.41	(2.38,63.66)	1.28	To observe one variable when the other variable was stratified	M
Zhang et al,1989b[14]	Years since last negative smear	0-2	1	(1,1)	18.17	To observe one variable when the other variable was stratified	M

Zhang et al,1989b[14]	Years since last negative smear	4-6	4.22	(1.51,11.9)	0.85	To observe one variable when the other variable was stratified	M
Cai et al,2008[12]	Interval since last Pap smear (years)	< 5	9.52	(2.23,49.54)	19.92	NA	M
Cai et al,2008[12]	Interval since last Pap smear (years)	≥ 5	4.75	(1.53,14.7)	2.13	NA	M
Cai et al,2008[12]	Interval since last Pap smear (years)	NA	1	(1,1)	77.96	NA	M

CI, confidence interval; LB, lower bound; M, Multivariate analysis; NA, Not applicable; OR, odds ratio; UB, Upper bound

Appendix 8. Study characteristics

Appendix Table 18. Study characteristics of the 11 included case-control studies

Study reference	Study period (years)	Age of patients (years)	Population sample size	
			Case	Control
**Systematic review on risk factors in Chinese women[1]**				
Zhang et al,1989a[11]	1973–1975	35–85	119	545
Zhang et al,1989b[14]	1974–1985	35–85	119	545
Peng et al,1991[7]	June 1987–November 1988	Case mean = 53.7, SD = 10.4<br>Controls mean = 51.7, SD = 10.8	101	146
Dong et al,1998[8]	August 1995–September 1996	25–70 (mean = 47)	43	327
Li et al,2000[9]	January 1989–May 1991	30–77	272	893
Cai et al,2008[12]	2003–2004	18+	110	110
Wang et al,2004[13]	2001–2002	Case = 24–78 (median: 51,39)<br>Control = 25–75 (median: 51,43)	129	143
Kan et al,2009[4]	September 2006–July 2008	Case = 21–85 (median: 46)<br>Control = 20–78 (median: 44.5)	893	1786
Li et al,2011[10]	September 2007–June 2010	Case = 28–60 (mean = 43.16, SD = 6.8)<br>Control = 29–58 (mean = 42.19, SD = 5.2)	112	200
**Updated review (3 additional papers)**				
Ye et al,2014[5]	June 2012 – May 2013	Case = 28-75, mean=50.38, SD=7.45; Control 29-72, mean=48.47, SD=6.89	120	120
Hu et al,2014[6]	March 2002 – December 2009	Case= 34-75, mean=51.8, SD=10.0; Control = 22=73, mean=42.5, SD=8.1	68	202

SD, standard deviation

Appendix Table 19. Study characteristics of the 3 studies excluded due to the risk of bias assessment based on initial results of the validation of the model

Study reference	Study period (years)	Age of patients (years)	Population sample size		Risk factor extracted to be included in meta-model
			Case	Control	
**Systematic review on risk factors in Chinese women[1]**					
Zhang and Xu,1990[15]	October 1987–November 1988		125	125	Number of sexual partners

Zhang et al,2010[16]	June–December 2004	20–78 (median: 43.2)	286	858	Smoking
**Updated review (3 additional papers)**					
Zhu et al,2016[17]	January 2012- December 2014	45-76 mean=57.7, SD=7.3	43	52	Smoking

SD, standard deviation

Appendix 9. Sensitivity analysis: impact of studies on model accuracy and precision

Appendix Figure 6. Impact of studies on absolute difference in predicted risk compared with base-case (model accuracy)

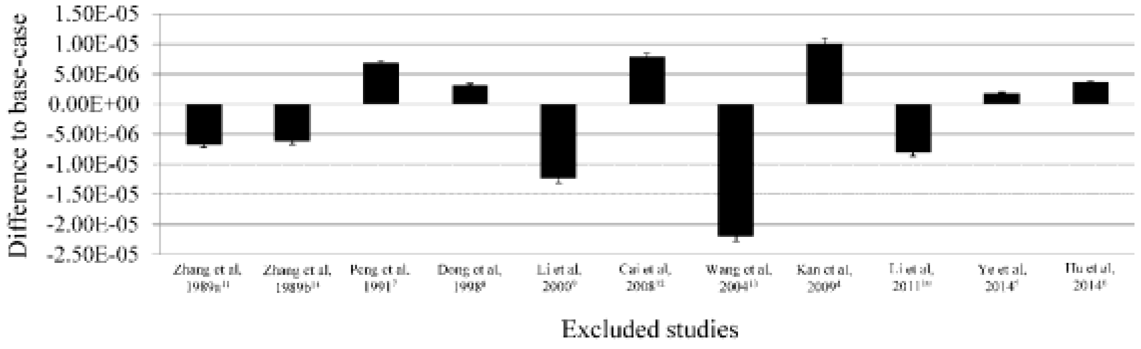

Appendix Figure 7. Impact of studies on difference in relative width of 95% confidence interval (CI) compared with base case (model precision)

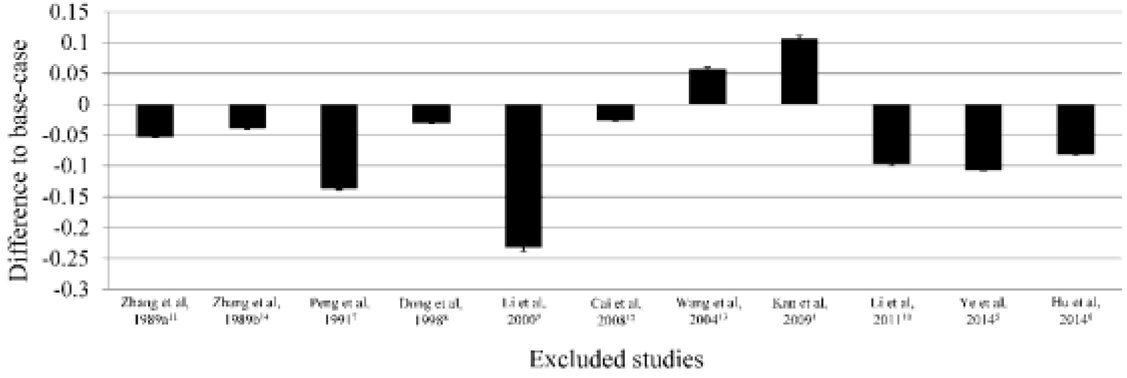

References

1. Li X, Hu SY, He Y, et al. Systematic literature review of risk factors for cervical cancer in the Chinese population. *Women's Health* 2018; 14: 1745506518816599. DOI: 10.1177/1745506518816599.

2. Borenstein M, V. Hedges L, Higgins J, et al. *Introduction to meta-analysis*. Chichester, West Sussex, U.K. ; Hoboken: John Wiley & Sons, 2009, p.online resource (xxviii, 421 pages).

3. Olsson U. Confidence Intervals for the Mean of a Log-Normal Distribution. *Journal of Statistics Education* 2005; 13: ww2.amstat.org/publications/jse/v13n11/olsson.html.

4. 阚士锋[Kan S], 芦鑫 [Lu X], 王传新 [Wang C], et al. 山东地区宫颈癌危险因素病例对照研究 [Risk factors of cervical cancer in Shandong Province: a case-control study]. *山东大学学报(医学版)* 2009; 47: 122-124.

5. 叶郁红[Ye Y], 张声[Zhang S], 王行富[Wang X], et al. 宫颈癌相关危险因素分析 [Risk Factors Analysis for Cervical Carcinoma]. *中国医科大学学报* 2014; 43: 659-660.

6. Hu B, Tao N, Zeng F, et al. A risk evaluation model of cervical cancer based on etiology and human leukocyte antigen allele susceptibility. *Int J Infect Dis* 2014; 28: 8-12. DOI: 10.1016/j.ijid.2014.05.015.

7. Peng HQ, Liu SL, Mann V, et al. Human papillomavirus types 16 and 33, herpes simplex virus type 2 and other risk factors for cervical cancer in Sichuan Province, China. *Int J Cancer* 1991; 47: 711-716.

8. Dong YZ, Sasagawa T, Fang SY, et al. Human papillomavirus, Chlamydia trachomatis, and other risk factors associated with cervical cancer in China. *Int J Clin Oncol* 1998; 3: 81-87.

9. Li HQ, Thomas DB, Jin SK, et al. Tubal sterilization and use of an IUD and risk of cervical cancer. *J Womens Health Gend Based Med* 2000; 9: 303-310. DOI: 10.1089/152460900318498.

10. 李健, 范岩峰, 许榕仙, et al. 宫颈癌相关影响因素病例对照研究 [Relationship between cervical cancer and risk factors: a case-control study]. *中国公共卫生* 2011; 27: 264-265.

11. Zhang ZF, Parkin DM, Yu SZ, et al. Risk factors for cancer of the cervix in a rural Chinese population. *Int J Cancer* 1989; 43: 762-767.

12. Cai HB, Ding XH, Zhou YF, et al. Risk factors for cervical cancer in China: a case-control study. *Eur J Gynaecol Oncol* 2008; 29: 72-75.

13. 王金桃, 高尔生, 程玉英, et al. 宫颈癌危险因素病例对照研究 [Case- control study on risk factors of cervical cancer]. *中国公共卫生* 2004: 37-38.

14. Zhang ZF, Parkin DM, Yu SZ, et al. Cervical screening attendance and its effectiveness in a rural population in China. *Cancer Detect Prev* 1989; 13: 337-342.

15. Zhang GN and Xu AQ. [Conditional logistic regression analysis and path analysis of risk factors of cervical cancer]. *Zhonghua liu xing bing xue za zhi = Zhonghua liuxingbingxue zazhi* 1990; 11: 212-216. 1990/08/01.

16. Zhang S, Zhao Q, Wang T, et al. Analysis of Cervical Cancer Risk Factor in Beijing 1: 3 Case-Control Epidemiological Survey. *Maternal and Child Health Care China* 2010; 25: 947-949.

17. 朱学英 [Zhu X], 熊向莉 [Xiong X] and 张琴芬[Zhang Q]. 绝经后阴道出血合并宫颈癌危险因素的Logistic回归分析 [Logistic regression analysis of risk factors in postmenopausal vaginal bleeding complicating cervical cancer]. *重庆医学* 2016; 3: 1032-1036.
